# Supplementary material for: A redox-mediated Kemp eliminase
Source: Nat Commun. 2017 Mar 28;8:14876. doi: 10.1038/ncomms14876 (PMC5379065; doi:10.1038/ncomms14876)
Supplement: Supplementary Information — Supplementary Figures, Supplementary Tables, Supplementary Methods and Supplementary References. [file ncomms14876-s1.pdf]

Supplementary Figures

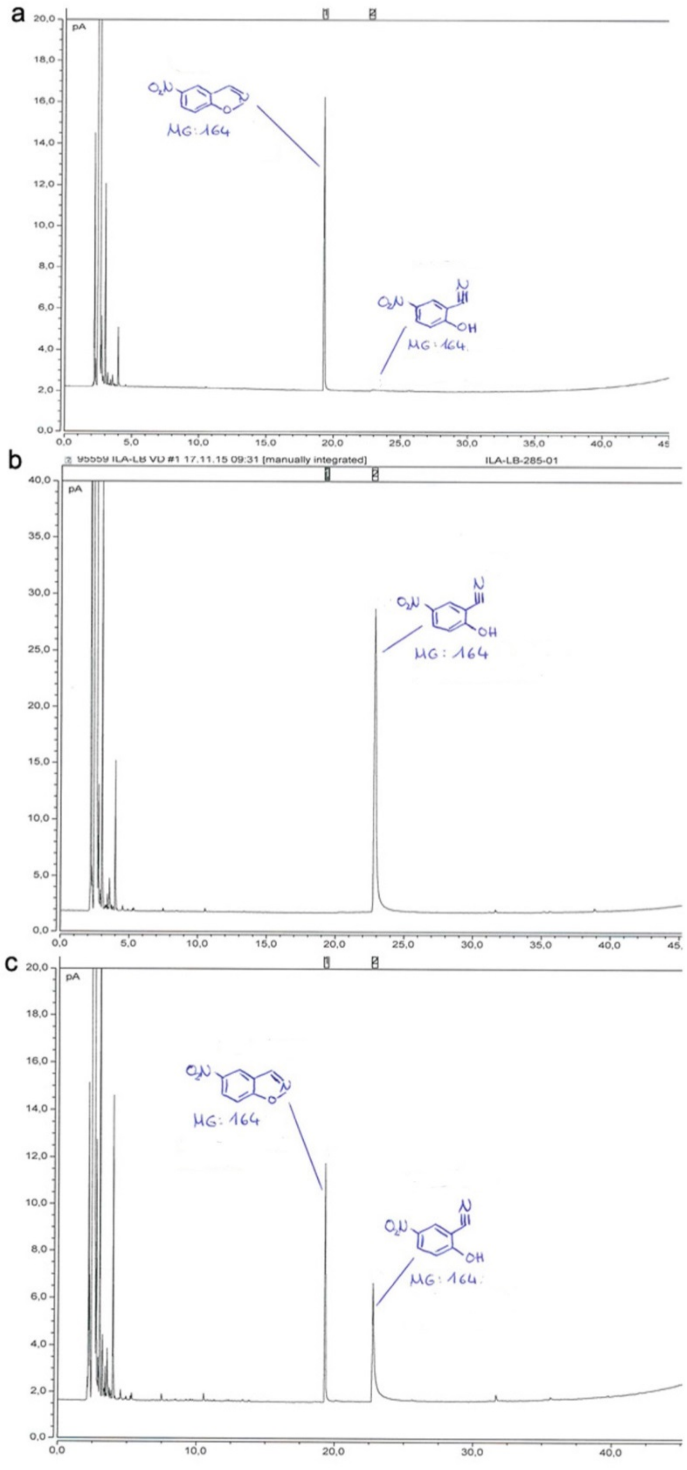

Supplementary Figure 1. Gas chromatography (GC) chromatograms. (**a**) Substrate standard. (**b**) Product standard. (**c**) Kemp elimination catalysed by purified wild type P450-BM3.

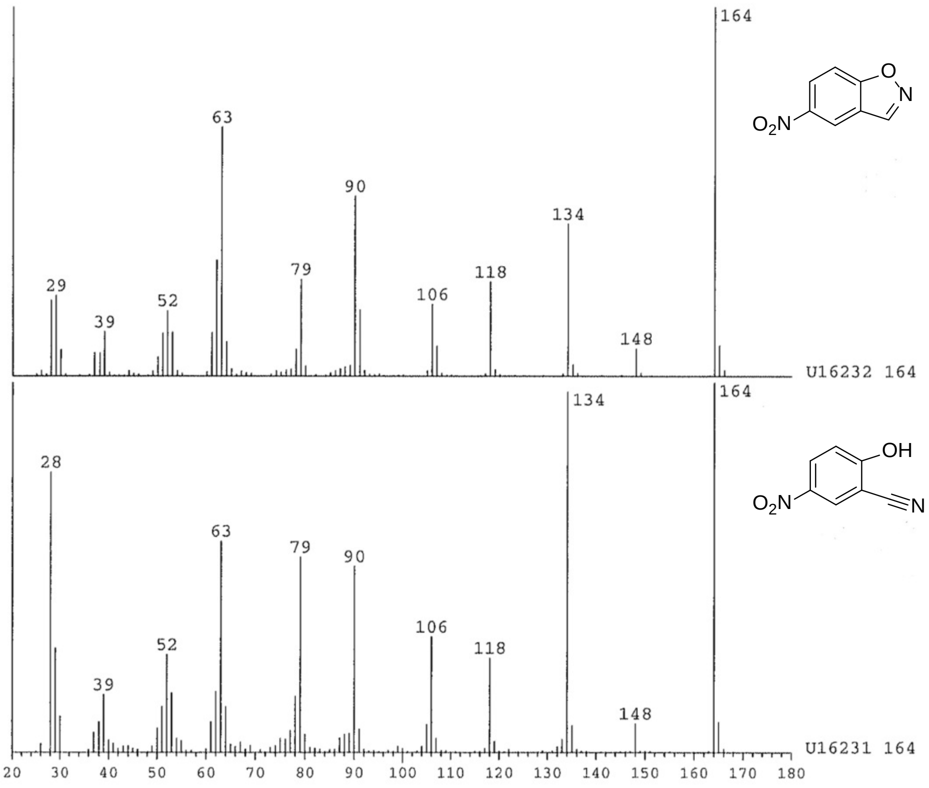

Supplementary Figure 2. Mass spectrometry (MS) analysis. MS spectrum for both substrate and product catalysed by purified WT P450BM-3.

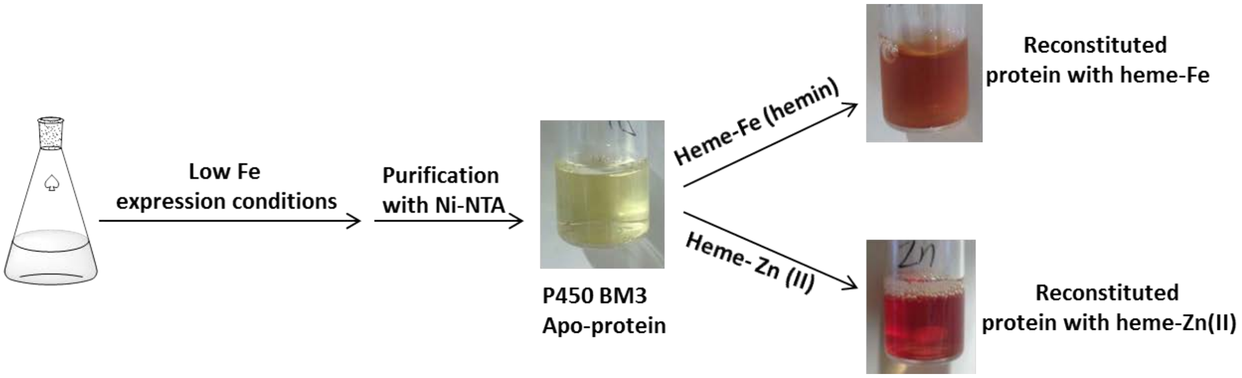

Supplementary Figure 3. Construction of reconstituted protein. Strategy for preparation of reconstituted protein containing heme-Fe (hemin) or heme-Zn(II) based on scaffold of P450-BM3 wild type.

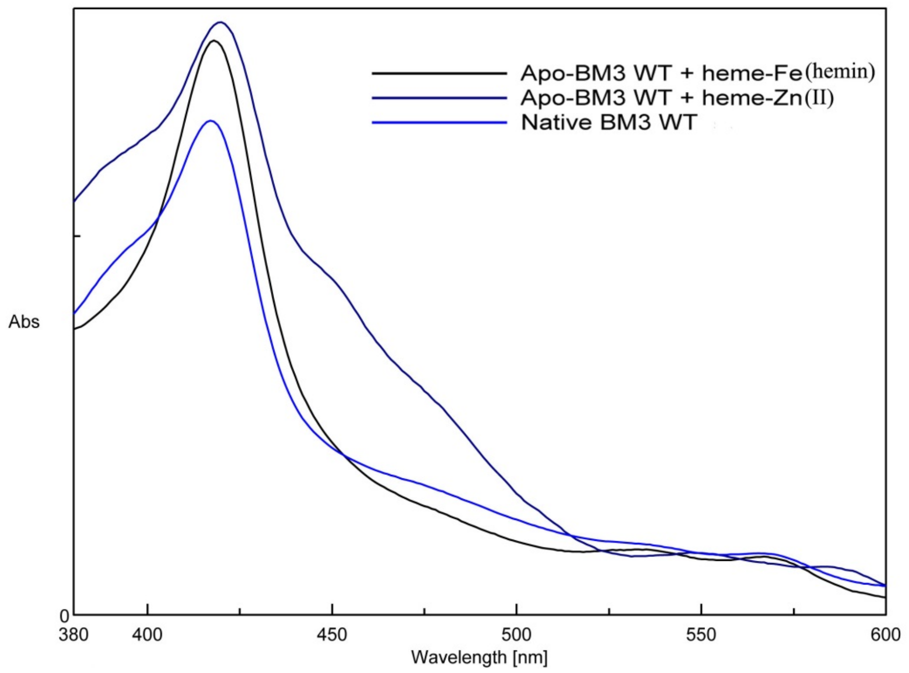

Supplementary Figure 4. **UltraViolet (UV) spectra analysis**. UV spectra of native and artificially reconstituted P450-BM3 proteins.

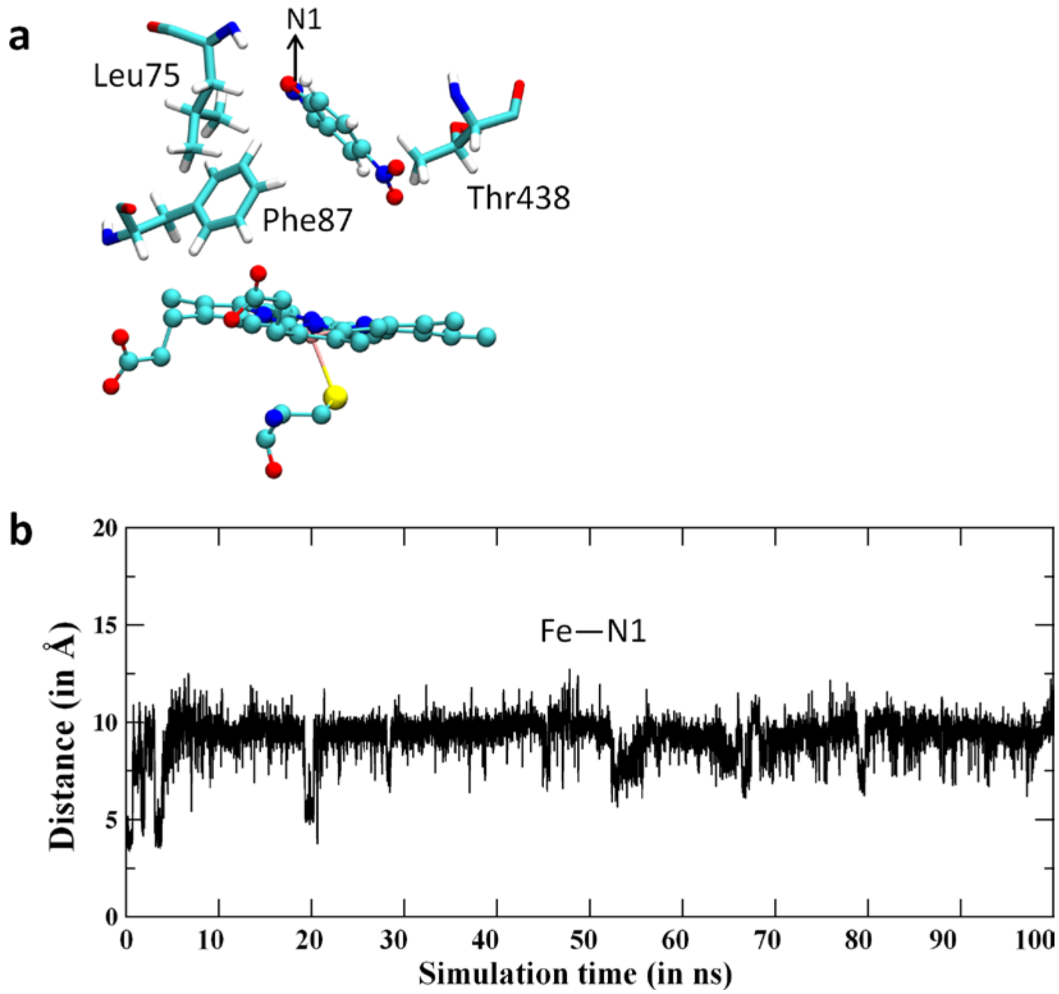

Supplementary Figure 5. MD simulation of substrate 1 with P450-BM3 WT in the Fe(III) resting state. (**a**) The representative snapshot in the equilibrium MD trajectory showing the active site structure of the WT in the Fe(III) resting state. (**b**) The distance fluctuations of Fe-N1 (the distance between the Fe center and substrate N1) for the WT in the Fe(III) resting state.

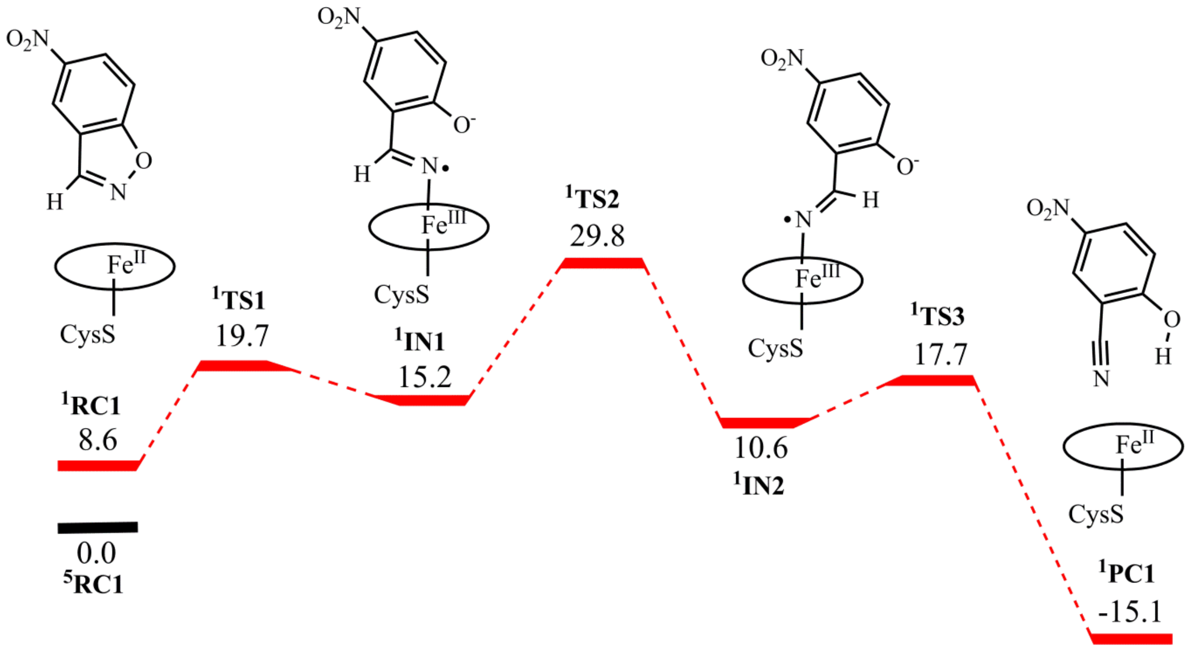

Supplementary Figure 6. QM/MM (UB3LYP/B2) relative energies (kcal/mol) for the redox-mediated Kemp elimination of 1 in the singlet state. All values are dispersion-corrected.

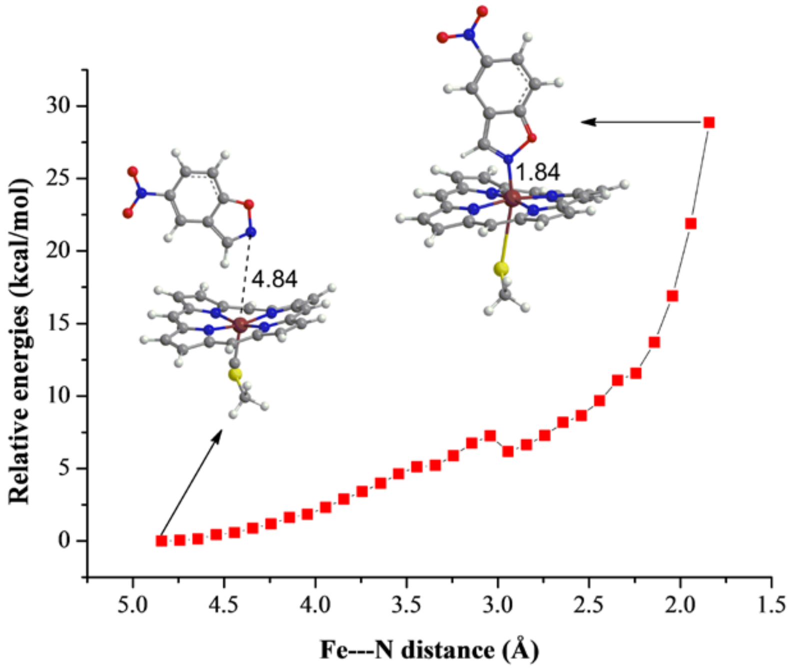

Supplementary Figure 7. QM/MM (UB3LYP/B1) scanned energy profile for Fe(II) in triplet state. QM/MM (UB3LYP/B1) scanned energy profile (kcal/mol) for the substrate N attack onto the Fe(II) of Fe(II)-heme in the triplet state.

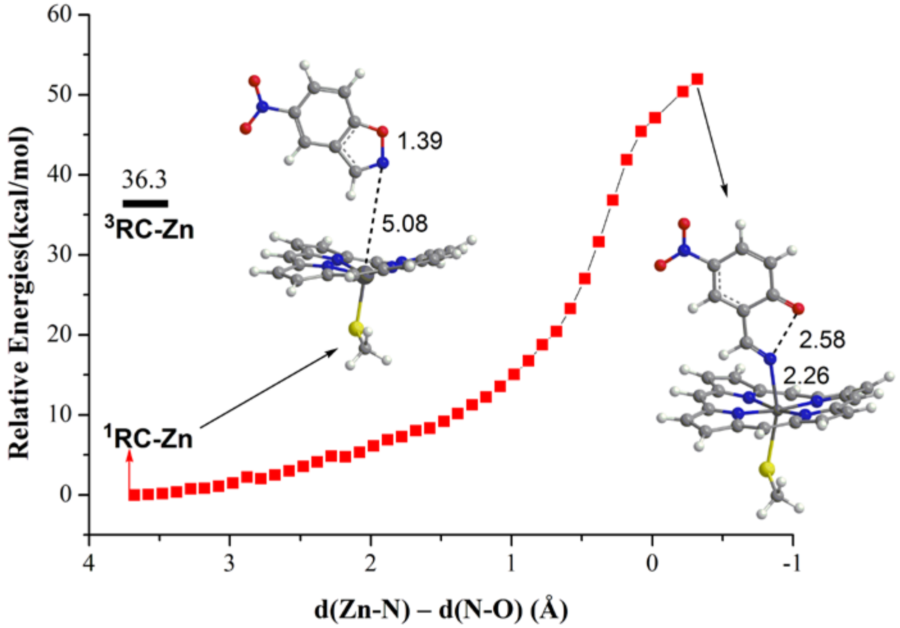

Supplementary Figure 8. QM/MM (UB3LYP/B1) scanned energy profile for Zn(II) in singlet state. QM/MM (UB3LYP/B1) scanned energy profile (kcal/mol) for the substrate N attack onto the Zn(II) of Zn(II)-heme in the singlet ground state.

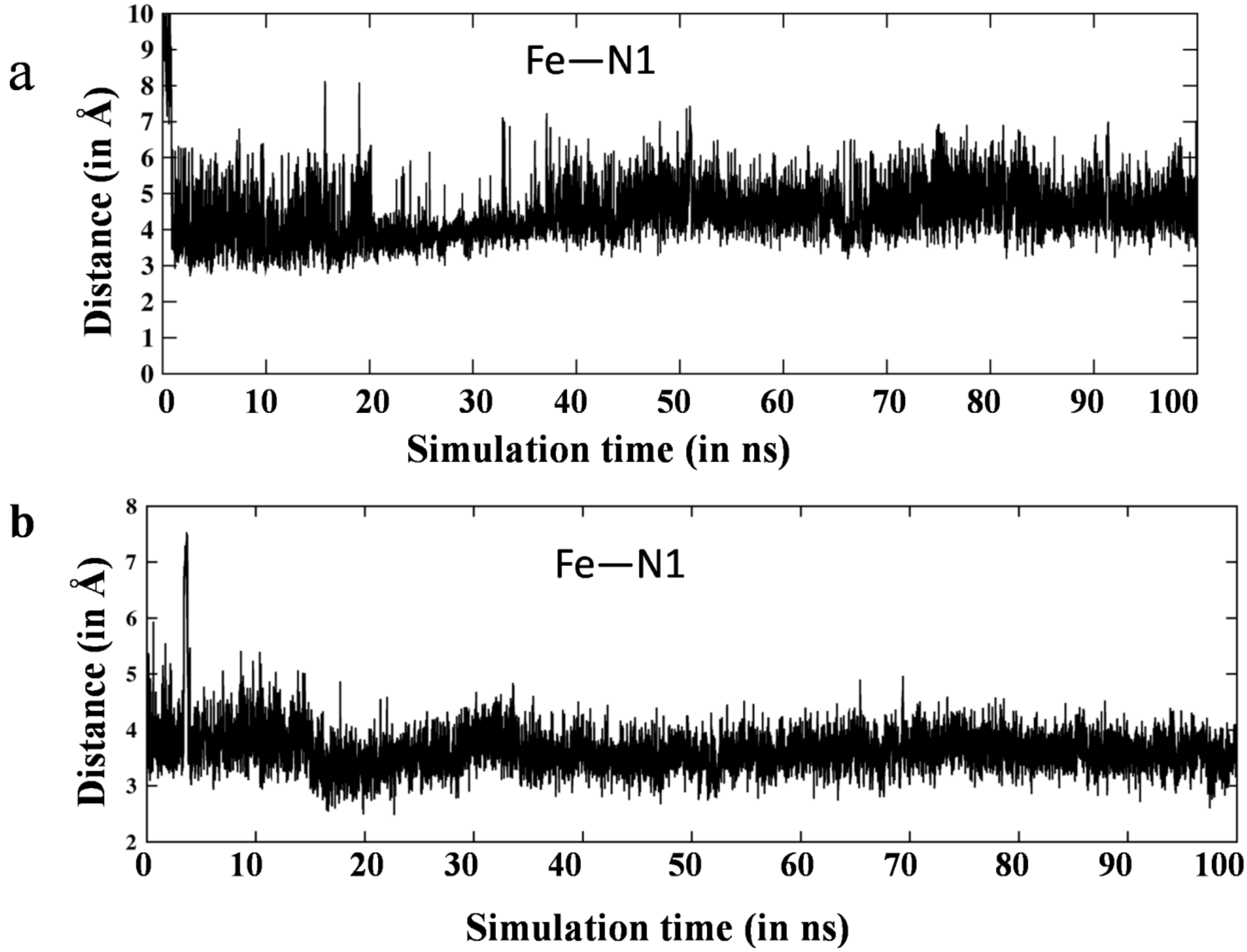

Supplementary Figure 9. Distance measurement between the Fe center and substrate N1. The distance fluctuations of Fe-N1(the distance between the Fe center and substrate N1) for: (**a**) WT, and (**b**) the variant F87G.

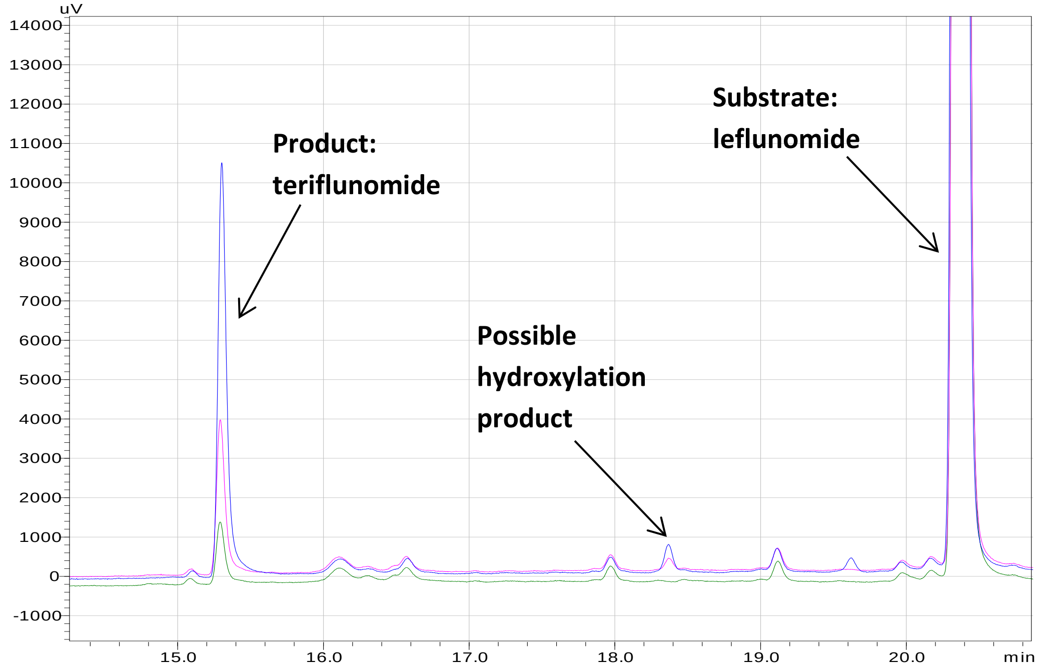

Supplementary Figure 10. Representative HPLC chromatograms for WT P450-BM3 and variant catalyzed isoxazole ring scission of leflunomide to form Kemp product teriflunomide (A771226). Green line: control reaction without enzyme addition; Pink line: reaction catalyzed by WT P450-BM3; blue line: reaction catalyzed by variant F87G/A82F.

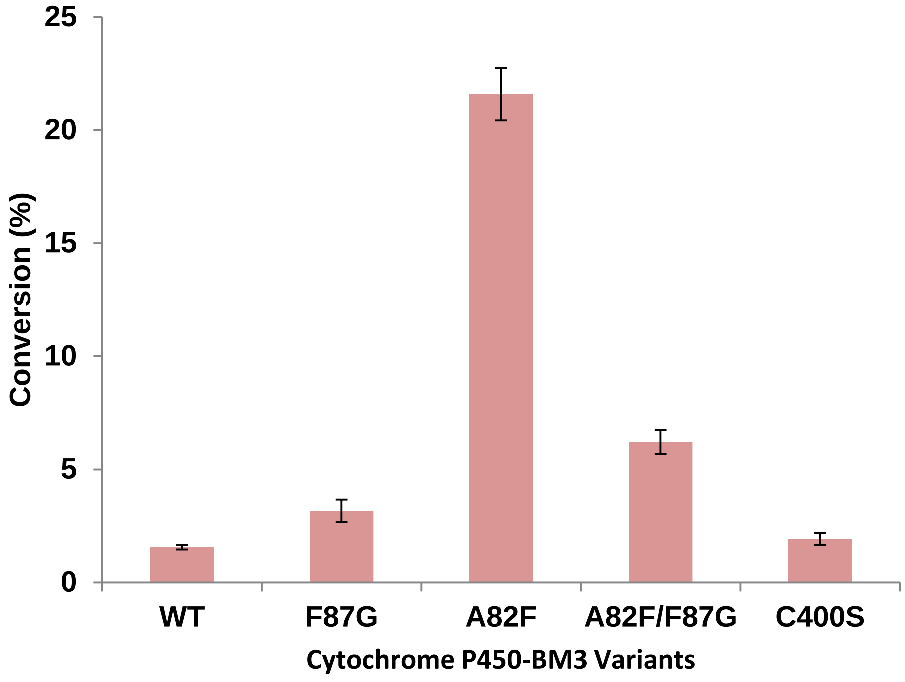

Supplementary Figure 11. Conversion of leflunomide to its Kemp product teriflunomide catalyzed by WT P450-BM3 and variants. Reaction conditions: 500 µL reaction consisting of 1 µM enzyme, 500 µM substrate, 0.25 mM $NADP^+$, 5 U/mL glucose dehydrogenase and 2 wt% glucose in phosphate buffer (50 mM, pH 8.0, 100 mM NaCl), 25°C, 1000 rpm for 5 h.

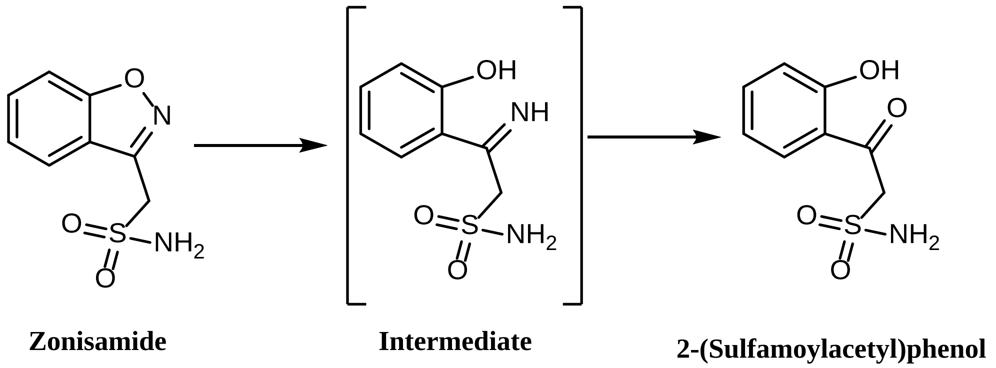

Supplementary Figure 12. Metabolism of zonisamide with human P450. Reductive isoxazole ring opening of zonisamide with P450 in human liver microsomes.

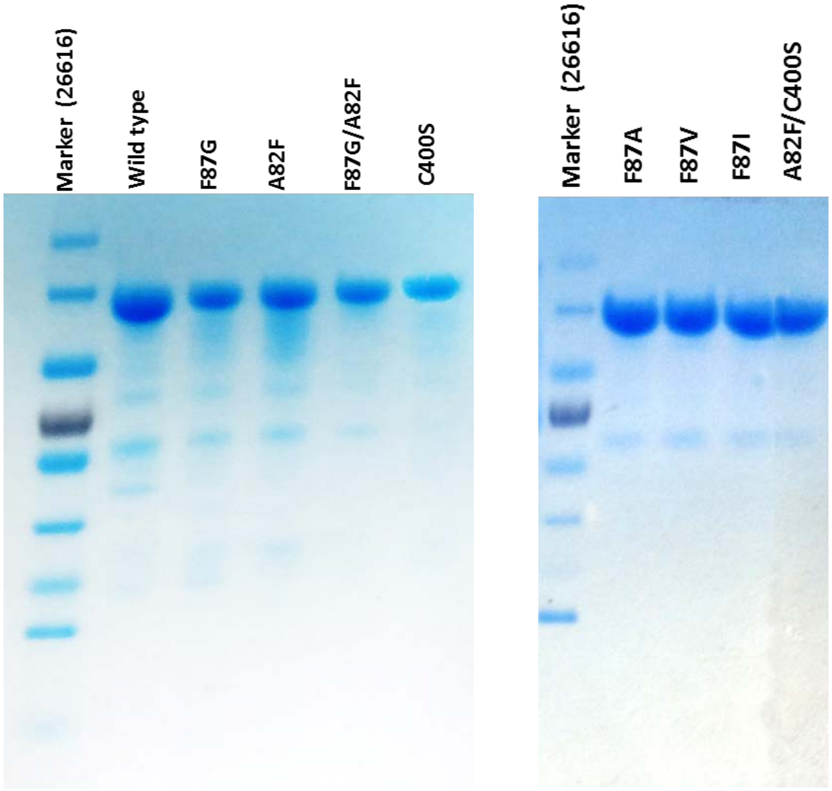

Supplementary Figure 13. SDS-PAGE analysis for the Kemp eliminases purified with Ni-NTA/His-tag column. The prestained protein ladder is from ThermoFisher Scientific (catalogue number 26616).

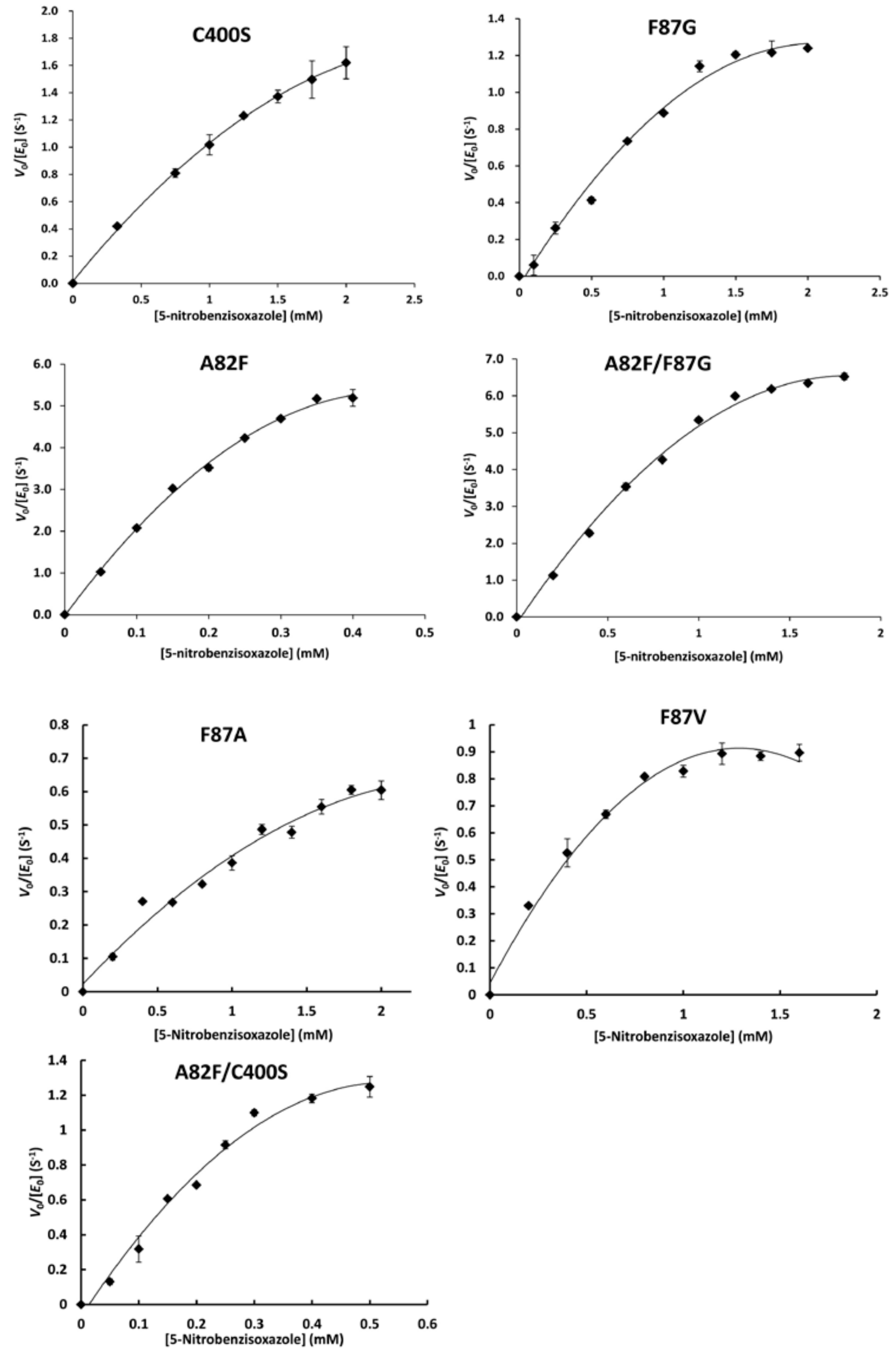

Supplementary Figure 14. Michaelis-Menten plots for representative Kemp eliminases for cleavage of 5-nitrobenzisoxazole 1. The data represent the average of three independent measurements, with error bars denoting s.d. Note the difference in the scale of specific product formation rate and the substrate concentration.

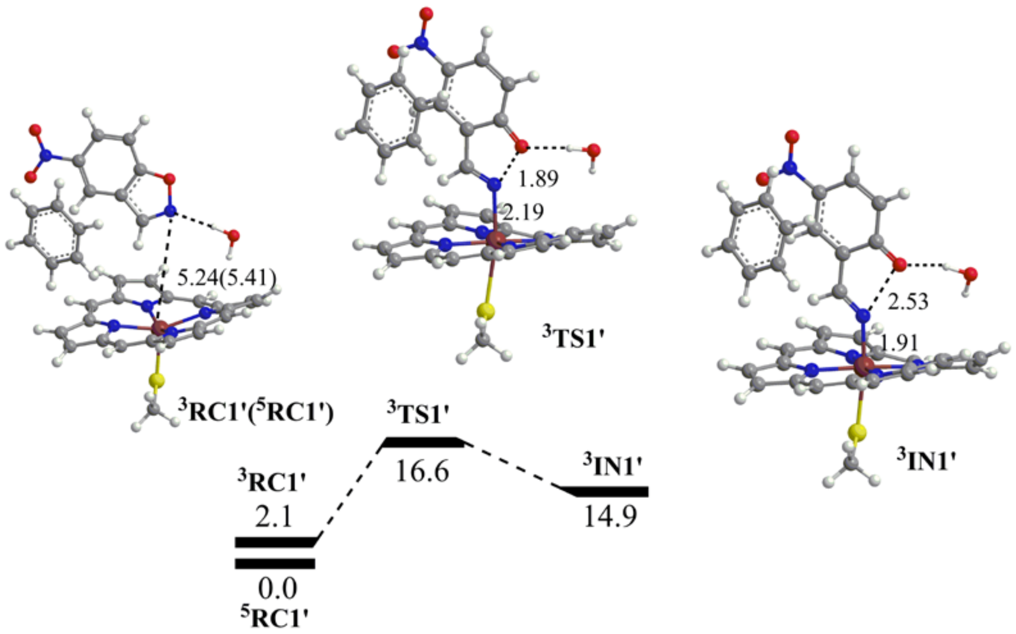

Supplementary Figure 15. QM/MM (UB3LYP/B2) relative energies (kcal/mol) for the first reaction step of Kemp elimination of 1 with a larger QM region. All values are dispersion-corrected. The QM/MM (UB3LYP/B1)-optimized QM structures are also shown along. The key distances are given in Å.

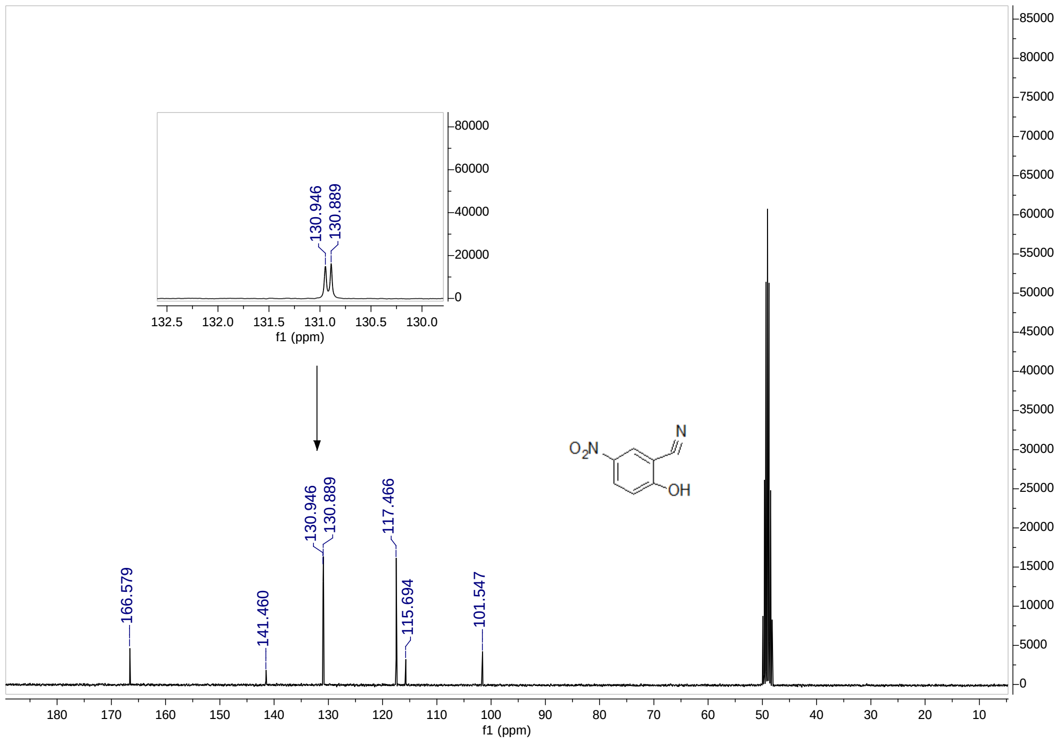

Supplementary Figure 16. ^{13}C NMR (75 MHz, $CDCl_3$) spectrum of product **2**.

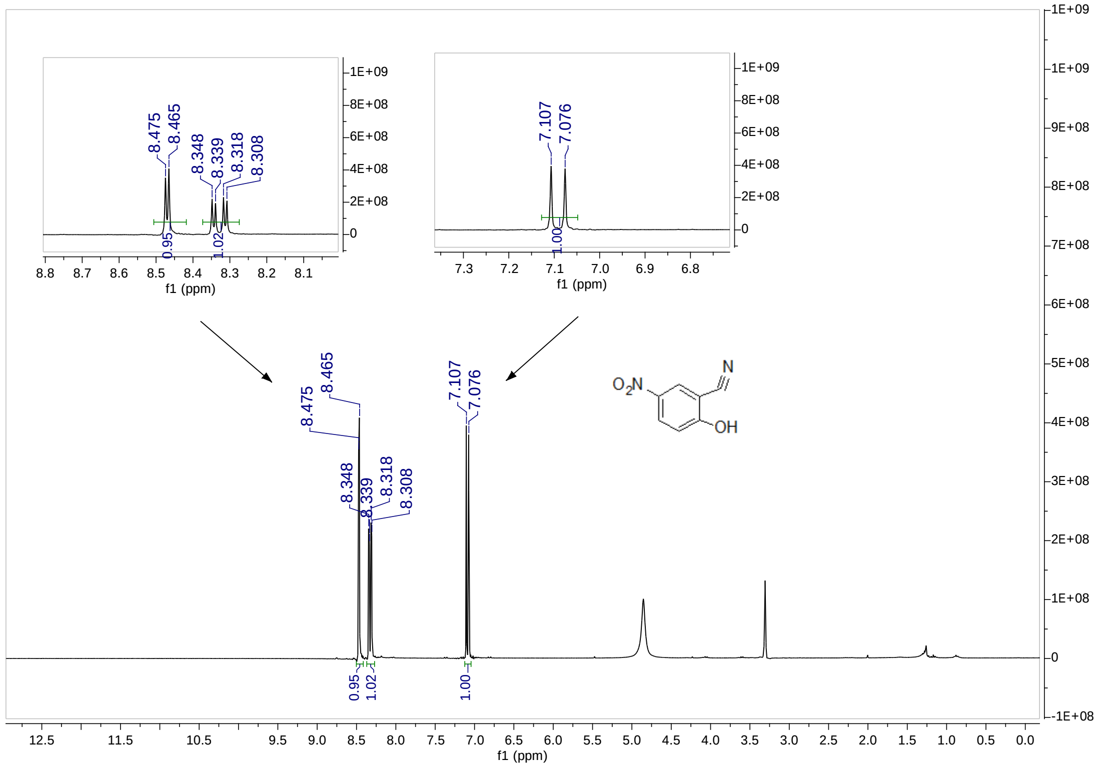

Supplementary Figure 17. ^{1}H NMR (300 MHz, $CDCl_3$) spectrum of product **2**.

Supplementary Tables

Supplementary Table 1. Kemp elimination catalyzed by purified wild type P450-BM3 under different conditions.

**Conditions**[*]	$\boldsymbol{v_o/[E_0]}$ **(s^{-1})**	**Relative activity (%)**
Enzyme+NADPH+Substrate	0.46 ± 0.01	100
Enzyme+Substrate	0.04[†]	N.A.
NADPH+Substrate	-[‡]	N.A.
Substrate	-[‡]	N.A.
Enzyme+NADPH+Substrate+CO	0.052±0.003	10

[*]Reaction conditions: 1 mL phosphate buffer (50 mM, pH 8.0, 100 mM NaCl), 1 µM enzyme, 5% acetonitrile as co-solvent, 0.5 mM substrate, NADPH 0.25 mM, 25°C, 380 nm. [†]Value detected due to the buffer catalyzed reaction. [‡]No activity detected. N.A.- not available

Supplementary Table 2. Kemp elimination catalyzed by wild type P450-BM3 under both aerobic and anaerobic conditions.

**Enzymes**	**Conditions***	$\boldsymbol{v_o/[E_0]}$ **(s^{-1})**
**WT**	Anaerobic	0.74 ± 0.01
	Aerobic	0.46 ± 0.01

*Reaction conditions: 1 mL phosphate buffer (50 mM, pH 8.0, 100 mM NaCl), enzyme: 1 µM, 5% acetonitrile as co-solvent, 0.5 mM substrate, NADPH 0.25 mM, 25°C, 380 nm.

Supplementary Table 3. Kemp elimination of substrate **1** catalyzed by wild type P450-BM3 and reconstituted proteins containing heme-Fe or heme Zn (II).

Enzymes*	$v_o/[E_0]$ (s^{-1})
**Native P450-BM3 WT**	0.46 ± 0.01
**Apo-P450-BM3 WT**	0.007 ± 0.002†
**Apo-P450-BM3 WT + Heme-Fe**	0.51 ± 0.01
**Apo-P450-BM3 WT + Heme-Zn (II)**	0.006 ± 0.001†

*Reaction conditions: 1 mL phosphate buffer (50 mM, pH 8.0, 100 mM NaCl), enzyme: 1 µM, 5% ACN as co-solvent, 0.5 mM substrate, NADPH 0.25 mM, 25°C, 380 nm.

†Extremely low activity detected due to small amount of heme-Fe in the apo-protein.

Supplementary Table 4. List of primers designed for variants creation.

template	mutants	Primers	Sequence (5’ to 3’)
WT	F87G	Forwards	GCAGGAGACGGGTTAGGAACAAGCTGGACGCATG
		Reverse	CATGCGTCCAGCTTGTTCCTAACCCGTCTCCTGC
WT	F87A	Forwards	GGAGACGGGTTAGCGACAAGCTGGACGCATG
		Reverse	CATGCGTCCAGCTTGTCGCTAACCCGTCTCC
WT	F87V	Forwards	GGAGACGGGTTAGTGACAAGCTGGACGCATG
		Reverse	CATGCGTCCAGCTTGTCACTAACCCGTCTCC
WT	F87I	Forwards	GGAGACGGGTTAATTACAAGCTGGACGCATG
		Reverse	CATGCGTCCAGCTTGTAATTAACCCGTCTCC
WT	A82F	Forwards	AAATTTGTACGTGATTTTTTTGGAGACGGGTTATTTAC
		Reverse	GTAAATAACCCGTCTCCAAAAAAATCACGTACAAATTT
WT	C400S	Forwards	GGAAACGGTCAGCGTGCGAGCATCGGTCAGCAGTTCGCTC
		Reverse	GAGCGAACTGCTGACCGATGCTCGCACGCTGACCGTTTCC
F87G	F87G/A82F	Forwards	AAATTTGTACGTGATTTTTTTGGAGACGGGTTAGGAAC
		Reverse	GTTCCTAACCCGTCTCCAAAAAAATCACGTACAAATTT
A82F	A82F/C400S	Forwards	GGAAACGGTCAGCGTGCGAGCATCGGTCAGCAGTTCGCTC
		Reverse	GAGCGAACTGCTGACCGATGCTCGCACGCTGACCGTTTCC

Supplementary Methods

System preparation and setup for computational analysis

The initial structures of P450-BM3 were taken from PDB code of 1JPZ[1]. The substrate 5-nitrobenzisoxazole was docked into the active site of P450-BM3 using AutoDock Vina tool[2] in Chimera[3]. Missing hydrogen atoms were added by module leap of Amber 14[4]. The force field for the heme moiety in the resting state (Fe(III)) was taken from the literature[5], while the force field for the one-electron reduced state (Fe(II)) was parameterized using "MCPB.py"[6]. The general AMBER force field (GAFF)[7] was used for the substrate 5-nitrobenzisoxazole, while the partial atomic charges and missing parameters were obtained from the RESP method[8,9], using HF/6-31G* level of theory. 15 Na^+ ions were added into the protein surface to neutralize the total charges of the systems. Finally, the resulting system was solvated in a rectangular box of TIP3P[10] waters extending up to minimum cutoff of 10 Å from the protein boundary. The Amber ff14SB force field[11] was employed for the protein in all of the Molecular Dynamics (MD) simulations

Computational details for **MD simulations**

After proper parameterizations and setup, the resulting system's geometries were minimized (5,000 steps for steepest conjugate and 10,000 steps for conjugate gradient) to remove poor contacts and relax the system. The systems were then annealed from 10 to 300 K under the constant amount of substance (N), volume (V) and temperature (T) (NVT ensemble) for 50 ps with a weak restraint of 5 kcal mol^{-1} $(Å^2)^{-1}$. Subsequently, the systems were maintained for 1 ns of density equilibration under constant amount of substance (N), pressure (P) and temperature (T), i.e. isothermal-isobaric (NPT ensemble) at target temperature of 300 K and the target pressure of 1.0 atm using Langevin-thermostat[12]and Barendsen barostat[13] with collision frequency of 2 ps and pressure relaxation time of 1 ps, with a weak restraint of 1 kcal mol^{-1} $(Å^2)^{-1}$. This 1 ns of density equilibration is not identical with conformational equilibration, but rather a weakly restrained MD in which we slowly relax the system to achieve a uniform density after heating dynamics under periodic boundary conditions. Thereafter, we removed all restraints applied during heating and density dynamics and further equilibrated the systems for ∼3 ns to get well settled pressure and temperature for conformational and chemical analyses. This was followed by a productive MD run, for each system, for 100 ns. During all MD simulations, the covalent bonds containing hydrogen were constrained using SHAKE[14], and particle mesh Ewald (PME)[15] was used to treat long-range electrostatic interactions. All MD simulations were performed with GPU version[16] of Amber 14 package.

Computational details for **QM/MM calculations**

Equilibrated snapshots from the MD simulations were taken for the subsequent QM/MM calculations. All the water molecules beyond the sphere of enzyme were removed. The resulting enzyme was solvated with a 16 Å layer of TIP3P water molecules (yielding a total of *ca.* 35,000 atoms). All QM/MM calculations were performed using ChemShell[17,18], combining Turbomole[19] for the QM part and DL_POLY[20] for the MM part. The CHARMM27 force field[21] was employed for the MM region. The electronic embedding scheme[22] was used to account for the polarizing effect of the enzyme environment on the QM region. Hydrogen link atoms with the charge-shift model[18] were applied to treat the QM/MM boundary. The QM region in our QM/MM calculations contained 58 atoms, including the heme, without side chains, the coordinating cysteine and the substrate. For the surrounding standard amino acid residues, these are lying away from the substrate and do not form strong electronic interactions (such as salt bridge, strong H-bonding interactions or involving electron transfer) with the selected QM region (heme moiety and the substrate), so that the standard MM charge and force field are sufficient for our QM/MM study. In QM/MM geometry optimizations, the QM region was treated by the hybrid UB3LYP[23,24] functional with two basis sets. For geometry optimization and frequency calculations the all electron basis set of def2-SVP[25], referred to as B1, was used. The energies are further corrected with the large all-electron basis-set Def2-TZVP[25], labeled as B2. All the QM/MM transition states (TSs) were located by relaxed potential energy surface (PES) scans followed by full TS optimizations using the P-RFO optimizer implemented in the HDLC code[26]. The empirical dispersion energy correction was calculated forall species by using the DFT-D3 program[27].

Chemicals

All chemicals were purchased from Sigma-Aldrich, Tokyo Chemical Industry (TCI) or Alfa Aesar and used without further purification. NMR spectra were recorded on a Bruker Avance 300 (^{1}H: 300 MHz, ^{13}C: 75 MHz) spectrometer using TMS as internal standard (d=0). Compound **2** was prepared according to a published protocol[28]. ^{1}H NMR (300 MHz, $CDCl_3$) δ 8.47 (d, *J* = 2.8 Hz, 1H), 8.33 (dd, *J* = 9.2, 2.8 Hz, 1H), 7.09 (d, *J* = 9.2 Hz, 1H). ^{13}C NMR (75 MHz, $CDCl_3$) δ 166.58 (s), 141.46 (s), 130.94 (s), 130. 88 (s), 117.47 (s), 115.69 (s), 101.55 (s).

GC-MS analysis

The reactions were initiated by addition of 1 mM NADPH in 500 µL sodium phosphate buffer containing P450-BM3 WT (1 µM) and 2 mM substrate **1** at 25°C, 800 rpm for 10 min. After reaction, HCl was added to adjust the pH value to 1-2 until the solution became colorless. The product was extracted with dichloromethane (0.5 mL X 2), the organic phase was separated by centrifugation, and dried over Na_2SO_4. The solvent was completely evaporated, followed by addition of 100 µL acetonitrile and then subjected to GC-MS analysis for product identification.

The analysis was conducted using an achiral column (Rtx-1, 29.5 m× 0.25 mm ×0.25 mm), with injector and detector temperatures at 230°C and 350°C, respectively. Temperature program: 60°C to 340°C at 5°C min^{-1}, then hold at 340°C for 10 min. The retention time for 5-nitro-benzisoxazole and its Kemp product are 19 and 23 min, respectively.

Supplementary References

1. Haines, D. C., Tomchick, D. R., Machius, M. & Peterson, J. A. Pivotal role of water in the mechanism of P450BM-3. *Biochemistry* **40**, 13456–13465 (2001).
2. Trott, O. & Olson, A. J. AutoDock Vina: improving the speed and accuracy of docking with a new scoring function, efficient optimization, and multithreading. *J Comput Chem*. ***31***, 455–461 (2010).
3. Pettersen, E. F. *et al*. UCSF Chimera—a visualization system for exploratory research and analysis. *J. Comp. Chem*. ***25***, 1605−1612 (2004).
4. Case, D. A. *et al*. AMBER 2015; University of California: San Francisco, 2015.
5. Shahrokh, K., Orendt, A., Yost, G. S. & Cheatham, T. E. Quantum mechanically derived AMBER-compatible heme parameters for various states of the cytochrome P450 catalytic cycle. *J. Comput. Chem.* **33**, 119–133 (2012).
6. Li, P. & Merz Jr, K. M. MCPB. py: A Python Based Metal Center Parameter Builder. *J. Chem. Inf. Model.* **56**, 599–604 (2016).
7. Wang, J., Wolf, R. M., Caldwell, J. W., Kollman, P. A. & Case, D. A. Development and testing of a general amber force field. *J. Comput. Chem*. ***25***, 1157–1174 (2004).
8. Bayly, C. I., Cieplak, P., Cornell, W. D. & Kollman, P. A. A Well-behaved electrostatic potential based method using charge restraints for deriving atomic charges: the RESP model. *J. Phys. Chem*. **97**, 10269–10280 (1993).
9. Cornell, W. D., Cieplak, P., Bayly, C. I. & Kollmann, P. A. Application of RESP charges to calculate conformational energies, hydrogen bond energies, and free energies of solvation. *J. Am. Chem. Soc*. **115**, 9620−9631 (1993).
10. Jorgensen, W. L., Chandrasekhar, J., Madura, J. D., Impey, R. W. & Klein, M. L. Comparison of simple potential functions for simulating liquid water. *J. Chem. Phys*., ***79***, 926−935 (1983).
11. Maier, J. A. *et al*. ff14sb: Improving the accuracy of protein side chain and backbone parameters from ff99sb. *J. Chem. Theory Comput*. ***11***, 696−3713 (2015).
12. Izaguirre, J. A., Catarello, D. P., Wozniak, J. M. & Skeel, R. D. Langevin stabilization of molecular dynamics. *J. Chem. Phys.* **114**, 2090–2098 (2001).
13. Berendsen, H. J., Postma, J. v., van Gunsteren, W. F., DiNola, A. & Haak, J. Molecular dynamics with coupling to an external bath. *J. Chem. Phys.* **81**, 3684–3690 (1984).
14. Ryckaert, J. -P., Ciccotti, G. & Berendsen, H. J. Numerical integration of the cartesian equations of motion of a system with constraints: molecular dynamics of n-alkanes. *J. Comput. Phys.* **23**, 327–341 (1977).
15. Darden, T., York, D. & Pedersen, L. Particle mesh Ewald: An N· log (N) method for Ewald sums in large systems. *J. Chem. Phys.* **98**, 10089–10092 (1993).
16. Salomon-Ferrer, R., Götz, A. W., Poole, D., Le Grand, S. & Walker, R. C. Routine microsecond molecular dynamics simulations with AMBER on GPUs. 2. Explicit solvent particle mesh Ewald. *J. Chem. Theor. Comput.* **9**, 3878–3888 (2013).
17. Sherwood, P. *et al*. QUASI: a general purpose implementation of the QM/MM approach and its application to problems in catalysis. *J. Mol. Struct.* **632**, 1–28 (2003).
18. Metz, S., Kästner, J., Sokol, A. A., Keal, T. W. & Sherwood, P. ChemShell—a modular software package for QM/MM simulations. *Comput. Mol. Sci.* **4**, 101–110 (2014).

19. Ahlrichs, R., Bär, M., Häser, M., Horn, H. & Kölmel, C. Electronic structure calculations on workstation computers: The program system turbomole. *Chem. Phys. Lett.* **162**, 165–169 (1989).
20. Smith, W. & Forester, T. DL_POLY_2. 0: A general-purpose parallel molecular dynamics simulation package. *J. Mol. Graph.* **14**, 136–141 (1996).
21. Brooks, B. R. *et al*. CHARMM: the biomolecular simulation program. *J. Comput. Chem*. ***30***, 1545–1614 (2009).
22. Bakowies, D. & Thiel, W. Hybrid models for combined quantum mechanical and molecular mechanical approaches. *J. Phys. Chem.* **100**, 10580–10594 (1996).
23. Lee, C. T., Yang, W. T. & Parr, R. G. Development of the Colle-Salvetti correlation-energy formula into a functional of the electrondensity. *Phys. Rev. B*. 37, 785−789 (1988).
24. Becke, A. D. Density-functional thermochemistry. III. The role of exact exchange. *J. Chem. Phys.* **98**, 5648–5652 (1993).
25. Weigend, F. & Ahlrichs, R. Balanced basis sets of split valence, triple zeta valence and quadruple zeta valence quality for H to Rn: design and assessment of accuracy. *Phys. Chem. Chem. Phys*. ***7***, 3297−3305 (2005).
26. Billeter, S. R., Turner, A. J. & Thiel, W. Linear scaling geometry optimisation and transition state search in hybrid delocalised internal coordinates. *Phys. Chem. Chem. Phys.* **2**, 2177–2186 (2000).
27. Grimme, S., Antony, J., Ehrlich, S. & Krieg, H. A consistent and accurate ab initio parametrization of density functional dispersion correction (DFT-D) for the 94 elements H-Pu. *J. Chem. Phys.* **132**, 154104 (2010).
28. Zamri, A., Schalk, I., Pattus, F. & Abdallah, M. Bacterial siderophores: synthesis and biological activities of novel pyochelin analogues. *Bioorganic & medicinal chemistry letters* **13**, 1147–1150 (2003).
