# Supplementary material for: A redox-mediated Kemp eliminase
Source: Nat Commun. 2017 Mar 28;8:14876. doi: 10.1038/ncomms14876 (PMC5379065; doi:10.1038/ncomms14876)
Supplement: Supplementary Data 1 — Cartesian coordinates of all species [file ncomms14876-s2.docx]

**Cartesian coordinates of all species**

**^5^RC1**

C 37.253412 35.234161 50.902306

H 38.067791 34.500648 50.976011

H 37.633836 36.155459 51.364102

S 36.795333 35.596120 49.167463

Fe 37.674431 37.791391 48.565473

N 39.117353 38.464453 49.989557

N 39.278188 37.485214 47.227861

N 36.405314 37.937648 46.846927

N 36.270780 38.958768 49.612247

C 38.846788 38.966291 51.236419

C 40.102737 39.252858 51.948421

C 41.118249 38.950619 51.072543

C 40.475444 38.475550 49.845897

C 40.611283 37.650753 47.462295

C 41.387425 37.193998 46.310256

C 40.484321 36.606992 45.436202

C 39.156859 36.908505 45.994038

C 36.715503 37.472649 45.607590

C 35.632401 37.764409 44.660856

C 34.642051 38.412885 45.383606

C 35.155079 38.485243 46.774799

C 34.966688 39.120056 49.236966

C 34.146801 39.391472 50.414411

C 34.995213 39.330050 51.504755

C 36.335538 39.115665 50.969306

C 37.541339 39.184275 51.707726

H 37.454926 39.563104 52.729587

C 41.155110 38.114912 48.674841

H 42.242897 38.195597 48.721085

C 37.943130 36.859732 45.282490

H 38.002453 36.421103 44.282083

C 34.489233 38.984421 47.915591

H 33.440766 39.271590 47.777000

C 37.390721 43.350047 46.943064

C 38.380969 44.094873 46.276300

C 38.156130 45.370708 45.741154

C 36.876242 45.884527 45.900730

C 35.873485 45.128778 46.552215

C 36.097940 43.863128 47.082951

C 38.088303 42.153648 47.326091

H 38.949814 45.926641 45.238579

H 36.620141 46.877372 45.530930

H 35.300465 43.325024 47.592383

H 37.725380 41.283703 47.877270

N 34.527671 45.710205 46.694110

O 33.634581 44.982002 47.107170

O 34.375355 46.884441 46.399332

N 39.324663 42.188700 46.918110

O 39.523781 43.395029 46.248325

h 36.519954 34.852222 51.612397

h 40.224996 39.642990 52.958825

h 42.197262 39.061925 51.179356

h 42.422109 37.485526 46.129960

h 40.635592 35.991390 44.549512

h 35.785222 37.569472 43.599389

h 33.689689 38.860464 45.099445

h 33.082048 39.621419 50.375773

h 34.752982 39.266792 52.565599

**^5^TS1**

C 37.241849 35.358959 50.799575

H 38.055787 34.622141 50.814740

H 37.646054 36.257795 51.288424

S 36.727582 35.798349 49.096893

Fe 37.663099 38.289602 48.459213

N 39.033682 38.596386 50.008151

N 39.204547 37.636560 47.216811

N 36.284228 38.019799 46.878086

N 36.131911 38.923801 49.716947

C 38.760327 39.042994 51.272822

C 40.020456 39.291557 51.987474

C 41.035760 39.005324 51.102401

C 40.392033 38.584291 49.861734

C 40.535633 37.784765 47.465071

C 41.314725 37.333265 46.309884

C 40.405135 36.789376 45.414579

C 39.075510 37.093434 45.971785

C 36.602721 37.598253 45.626957

C 35.473297 37.821472 44.722054

C 34.447343 38.362819 45.488183

C 34.989981 38.449894 46.864694

C 34.821155 39.029175 49.358029

C 34.017663 39.299440 50.549806

C 34.889971 39.289795 51.620238

C 36.229944 39.109805 51.062077

C 37.452575 39.221442 51.766207

H 37.379314 39.576499 52.797115

C 41.071561 38.236811 48.684227

H 42.159554 38.308045 48.739054

C 37.857139 37.054683 45.267828

H 37.916067 36.649598 44.253702

C 34.325237 38.879505 48.042101

H 33.257490 39.100189 47.934847

C 37.053647 42.509976 47.209973

C 38.466836 42.658449 47.090826

C 38.984901 43.926363 46.708565

C 38.107269 44.975947 46.498891

C 36.712161 44.795243 46.642141

C 36.171280 43.566272 47.002746

C 36.794096 41.153098 47.605422

H 40.059439 44.080972 46.620725

H 38.484513 45.963983 46.233352

H 35.095440 43.465573 47.140976

H 35.772242 40.781467 47.763812

N 35.807946 45.930481 46.472763

O 34.598804 45.714811 46.522904

O 36.298922 47.036317 46.299042

N 37.774690 40.351476 47.773838

O 39.147173 41.593890 47.359445

h 36.536210 34.953308 51.524550

h 40.155203 39.656172 53.005790

h 42.116146 39.095869 51.214802

h 42.361653 37.592428 46.152270

h 40.550448 36.194151 44.513103

h 35.608873 37.654327 43.653528

h 33.446778 38.718166 45.241880

h 32.948859 39.511652 50.523545

h 34.668606 39.219875 52.685214

**^5^IN1**

C 37.262179 35.404882 50.796725

H 38.074702 34.667765 50.802931

H 37.664501 36.300243 51.291328

S 36.762996 35.853772 49.091722

Fe 37.672739 38.317895 48.462250

N 39.061956 38.569241 49.989028

N 39.171801 37.621909 47.212295

N 36.270942 38.021611 46.927225

N 36.166107 38.918768 49.754769

C 38.812215 39.012884 51.265272

C 40.082591 39.275755 51.946422

C 41.081194 39.023372 51.030512

C 40.417787 38.596045 49.806230

C 40.509943 37.795888 47.415097

C 41.258647 37.314860 46.255707

C 40.334828 36.717417 45.413420

C 39.016825 37.036399 45.986718

C 36.554597 37.566159 45.678466

C 35.409761 37.788362 44.795200

C 34.414255 38.371957 45.568244

C 34.984486 38.484865 46.925818

C 34.855877 39.068304 49.410544

C 34.075896 39.358667 50.608204

C 34.959212 39.321081 51.669424

C 36.285704 39.104234 51.099119

C 37.518281 39.196452 51.785879

H 37.466563 39.563415 52.813634

C 41.073710 38.272692 48.608560

H 42.159916 38.375381 48.627768

C 37.787968 36.987088 45.307077

H 37.821379 36.558907 44.301822

C 34.341884 38.929765 48.103302

H 33.280479 39.178501 48.004533

C 37.076356 42.532225 47.226317

C 38.445276 42.882902 46.889200

C 38.649237 44.235580 46.414888

C 37.605529 45.124502 46.320604

C 36.284009 44.733263 46.672867

C 36.027527 43.444888 47.127276

C 36.891752 41.187924 47.708971

H 39.665784 44.538990 46.160967

H 37.771937 46.148137 45.982614

H 35.010869 43.177299 47.417052

H 35.854588 40.926995 47.991924

N 35.200269 45.688821 46.626823

O 34.055788 45.294215 46.870962

O 35.465213 46.856214 46.356809

N 37.784341 40.293650 47.834295

O 39.351765 42.028580 47.025679

h 36.551863 34.993929 51.514105

h 40.241516 39.621913 52.967688

h 42.159993 39.152607 51.117445

h 42.295566 37.585674 46.056896

h 40.465887 36.079977 44.539028

h 35.504247 37.599150 43.725930

h 33.414069 38.734069 45.330399

h 33.015289 39.609277 50.588875

h 34.742824 39.245897 52.735064

**^5^TS2**

C 37.266407 35.392901 50.814083

H 38.092386 34.672343 50.835667

H 37.639268 36.302848 51.302740

S 36.782787 35.809587 49.096186

Fe 37.652493 38.137007 48.441028

N 39.081739 38.520217 49.909300

N 39.147170 37.544093 47.147482

N 36.262892 37.941789 46.902152

N 36.222232 38.912278 49.719814

C 38.862551 38.923045 51.210549

C 40.149455 39.143587 51.874149

C 41.125843 38.913705 50.928085

C 40.437119 38.531912 49.706418

C 40.486614 37.742864 47.316989

C 41.217458 37.270513 46.146653

C 40.291520 36.638367 45.335282

C 38.979013 36.942860 45.927318

C 36.522998 37.480320 45.651368

C 35.386923 37.748470 44.774181

C 34.422231 38.380048 45.549188

C 35.005744 38.484188 46.900208

C 34.916915 39.114478 49.373057

C 34.144571 39.405869 50.575125

C 35.022217 39.315579 51.639334

C 36.341903 39.069603 51.068503

C 37.580365 39.118827 51.752984

H 37.546343 39.467698 52.788366

C 41.073192 38.220492 48.496181

H 42.159672 38.321754 48.493553

C 37.744428 36.883297 45.265489

H 37.762736 36.451599 44.261490

C 34.397106 38.989639 48.068842

H 33.351536 39.296480 47.969632

C 37.485099 42.580116 47.328782

C 38.580306 43.170321 46.551226

C 38.235326 44.418392 45.881979

C 36.984808 44.978316 45.950770

C 35.951174 44.328913 46.674604

C 36.213633 43.128890 47.353147

C 37.782827 41.382219 48.185974

H 39.035321 44.893751 45.308710

H 36.757907 45.919904 45.448940

H 35.408444 42.674641 47.931184

H 37.944125 41.523579 49.277703

N 34.642147 44.909601 46.750386

O 33.729516 44.249940 47.268235

O 34.464818 46.037325 46.288494

N 37.798933 40.232603 47.701012

O 39.713334 42.658522 46.481244

h 36.549016 34.975508 51.520624

h 40.340412 39.448974 52.902906

h 42.210067 39.007568 50.989031

h 42.243277 37.564369 45.924367

h 40.421361 35.985694 44.472017

h 35.477197 37.567615 43.703102

h 33.432719 38.770682 45.311833

h 33.089616 39.679487 50.558706

h 34.801642 39.242127 52.704237

**^5^IN2**

C 37.276950 35.374704 50.837621

H 38.103350 34.654630 50.880586

H 37.645455 36.289990 51.320481

S 36.818891 35.770665 49.107833

Fe 37.596874 38.183476 48.523092

N 39.027043 38.500320 50.004019

N 39.108591 37.626456 47.202938

N 36.210460 37.957298 46.973742

N 36.136439 38.810149 49.833183

C 38.795422 38.914389 51.296838

C 40.076834 39.170708 51.959774

C 41.060942 38.944123 51.021782

C 40.381069 38.541939 49.800266

C 40.448388 37.809361 47.389332

C 41.187305 37.345330 46.219245

C 40.261008 36.737734 45.387422

C 38.946438 37.037101 45.976362

C 36.479280 37.533884 45.712054

C 35.334925 37.795983 44.843179

C 34.354833 38.379194 45.637020

C 34.939860 38.464520 46.989821

C 34.838780 39.032860 49.480372

C 34.070485 39.353305 50.679180

C 34.948986 39.271461 51.741932

C 36.266687 39.003502 51.171919

C 37.509887 39.086417 51.843328

H 37.475089 39.454496 52.871761

C 41.024723 38.261421 48.585655

H 42.110882 38.367838 48.593875

C 37.711518 36.970653 45.313016

H 37.734893 36.557096 44.301437

C 34.323153 38.930045 48.172474

H 33.277473 39.236915 48.075528

C 37.720200 42.486476 46.939465

C 38.733564 43.304260 46.252308

C 38.280154 44.613757 45.812083

C 36.991705 45.035239 46.009172

C 36.038020 44.186527 46.642065

C 36.403350 42.924625 47.104383

C 38.243278 41.223773 47.425090

H 39.018811 45.251471 45.321035

H 36.666241 46.024243 45.684007

H 35.662206 42.311422 47.617018

H 39.351871 41.206184 47.277462

N 34.683493 44.648687 46.824194

O 33.840571 43.852542 47.250585

O 34.415612 45.814489 46.538447

N 37.682163 40.213105 47.913165

O 39.895232 42.888749 46.057530

h 36.555686 34.962889 51.543491

h 40.254328 39.509012 52.980613

h 42.143355 39.055116 51.086051

h 42.221630 37.621801 46.014786

h 40.389070 36.103416 44.510320

h 35.431190 37.650424 43.767255

h 33.352183 38.742356 45.411469

h 33.024151 39.657857 50.656795

h 34.732596 39.195841 52.807542

**^5^TS3**

C 37.250469 35.369667 50.811523

H 38.063220 34.632277 50.819642

H 37.652944 36.265477 51.305674

S 36.746066 35.818314 49.107764

Fe 37.585326 38.309464 48.501149

N 38.996574 38.532354 50.029875

N 39.094119 37.616680 47.225260

N 36.198324 38.038680 46.958938

N 36.108822 38.904148 49.815987

C 38.757810 38.958932 51.312137

C 40.036885 39.208431 51.986443

C 41.026714 38.953132 51.061766

C 40.350542 38.546258 49.837094

C 40.431187 37.785282 47.427339

C 41.177830 37.328177 46.256123

C 40.251534 36.750704 45.404189

C 38.934168 37.055599 45.990769

C 36.470868 37.594875 45.704815

C 35.320760 37.831317 44.833711

C 34.331325 38.405943 45.622188

C 34.918226 38.518021 46.973430

C 34.804678 39.086388 49.465583

C 34.023160 39.360008 50.669412

C 34.901648 39.284997 51.732359

C 36.228422 39.061995 51.160779

C 37.465489 39.137292 51.844430

H 37.420081 39.488177 52.878383

C 40.999343 38.236099 48.630400

H 42.087473 38.322443 48.651479

C 37.701880 37.019911 45.315487

H 37.727049 36.611017 44.301677

C 34.291605 38.973655 48.156739

H 33.235281 39.245128 48.062465

C 37.479390 42.598741 46.987601

C 38.529889 43.380027 46.370466

C 38.207760 44.689606 45.910986

C 36.922372 45.169168 46.072736

C 35.914423 44.372562 46.664783

C 36.183988 43.082317 47.131225

C 38.071759 41.319019 47.387013

H 38.988785 45.299089 45.452923

H 36.657611 46.175506 45.746286

H 35.397202 42.497825 47.606277

H 39.229072 41.660117 46.959117

N 34.575360 44.915376 46.814030

O 33.684992 44.161701 47.206447

O 34.388975 46.096297 46.542386

N 37.810619 40.233177 47.874311

O 39.686623 42.829449 46.271818

h 36.540903 34.958979 51.529798

h 40.204251 39.562114 53.003774

h 42.109662 39.050866 51.137518

h 42.213729 37.605167 46.060533

h 40.377263 36.136325 44.512675

h 35.418508 37.669148 43.760299

h 33.320235 38.746078 45.398420

h 32.966796 39.628065 50.651868

h 34.685871 39.196178 52.797074

**^5^PC1**

C 37.264968 35.205169 50.912305

H 38.080542 34.474215 51.004384

H 37.641310 36.132727 51.365698

S 36.822163 35.547379 49.169685

Fe 37.646184 37.793615 48.592673

N 39.066435 38.396937 50.061774

N 39.254383 37.481923 47.266402

N 36.369456 37.914969 46.884499

N 36.203716 38.850997 49.681342

C 38.785788 38.906089 51.300815

C 40.037381 39.217716 52.014001

C 41.059005 38.918464 51.145561

C 40.424333 38.430302 49.919096

C 40.579428 37.673393 47.513695

C 41.369304 37.302233 46.341709

C 40.478781 36.741370 45.431394

C 39.146494 36.969428 46.004478

C 36.688918 37.481560 45.635330

C 35.595224 37.765060 44.696569

C 34.594010 38.379761 45.432835

C 35.109266 38.437130 46.825056

C 34.905789 39.021703 49.299812

C 34.081870 39.299517 50.475339

C 34.927984 39.243498 51.566256

C 36.270083 39.021940 51.033148

C 37.476079 39.108972 51.769911

H 37.384590 39.494639 52.788865

C 41.111127 38.105598 48.744508

H 42.197789 38.202339 48.794593

C 37.931332 36.912755 45.292008

H 38.001165 36.505985 44.278847

C 34.434075 38.902127 47.975317

H 33.383441 39.182620 47.839149

C 37.604893 43.274610 46.765993

C 38.558752 43.969044 45.970432

C 38.237300 45.245853 45.468820

C 36.997222 45.805312 45.732979

C 36.048114 45.082657 46.475478

C 36.341634 43.828853 47.001797

C 38.000526 42.013965 47.314020

H 38.991799 45.776349 44.883695

H 36.741084 46.800874 45.371655

H 35.604297 43.301668 47.604500

H 39.912387 42.600456 46.093938

N 34.719546 45.658160 46.708586

O 33.849848 44.925118 47.163618

O 34.551072 46.835655 46.431407

N 38.423566 41.006545 47.708623

O 39.748044 43.458302 45.667393

h 36.528114 34.829657 51.622306

h 40.153087 39.628070 53.017140

h 42.136773 39.039450 51.254395

h 42.401826 37.616083 46.188531

h 40.649815 36.175875 44.515409

h 35.744578 37.586593 43.631717

h 33.630526 38.810078 45.159720

h 33.020398 39.543179 50.430929

h 34.687823 39.185369 52.627863

**^3^RC1**

C 37.254822 35.271961 50.888479

H 38.096211 34.572402 51.000824

H 37.590607 36.212785 51.348726

S 36.849671 35.597702 49.135355

Fe 37.711108 37.989081 48.493174

N 39.067165 38.498540 49.916344

N 39.222734 37.489823 47.241908

N 36.453795 37.965765 46.889075

N 36.279052 38.833391 49.624744

C 38.817824 38.945147 51.196201

C 40.070641 39.224560 51.901754

C 41.079624 38.958830 51.008206

C 40.432008 38.518378 49.778980

C 40.562600 37.679293 47.436023

C 41.331654 37.174466 46.302705

C 40.430710 36.518407 45.483011

C 39.110027 36.840519 46.041146

C 36.725809 37.455184 45.652303

C 35.645734 37.741617 44.709543

C 34.671700 38.418219 45.425626

C 35.190991 38.501850 46.807265

C 34.977563 39.040121 49.256552

C 34.158462 39.320313 50.429230

C 35.000678 39.242220 51.518871

C 36.334143 39.009165 50.982174

C 37.529850 39.108844 51.712693

H 37.452302 39.465288 52.742268

C 41.121882 38.178980 48.615541

H 42.208064 38.274032 48.655754

C 37.910155 36.779923 45.327971

H 37.946261 36.299092 44.346610

C 34.503196 38.964564 47.939374

H 33.458752 39.261201 47.799018

C 37.408990 43.416928 46.916743

C 38.393738 44.156757 46.237002

C 38.166048 45.433215 45.703452

C 36.888233 45.949596 45.873980

C 35.889655 45.195867 46.534525

C 36.117606 43.931396 47.066038

C 38.109277 42.222931 47.300631

H 38.954714 45.988372 45.191581

H 36.629849 46.942840 45.506689

H 35.324516 43.395320 47.584580

H 37.751977 41.359127 47.864543

N 34.544775 45.777506 46.687907

O 33.657391 45.050762 47.115952

O 34.387486 46.949078 46.386133

N 39.341541 42.252511 46.879774

O 39.535552 43.454650 46.201627

h 36.529173 34.872910 51.597181

h 40.202946 39.592879 52.919056

h 42.159587 39.063884 51.111698

h 42.360229 37.469798 46.095630

h 40.575800 35.859310 44.627087

h 35.798430 37.540225 43.649264

h 33.725989 38.876124 45.135761

h 33.097875 39.568355 50.388040

h 34.759732 39.194986 52.580841

**^3^TS1**

C 37.166884 35.226957 50.903823

H 37.975176 34.479579 50.952110

H 37.583419 36.123028 51.392798

S 36.679322 35.660405 49.197808

Fe 37.732063 38.658308 48.409449

N 39.087864 38.894458 49.905929

N 39.209688 37.965062 47.219748

N 36.363805 38.289799 46.933547

N 36.239418 39.094930 49.670373

C 38.830797 39.237233 51.206718

C 40.080814 39.474214 51.928073

C 41.096610 39.277155 51.020858

C 40.450626 38.924044 49.765888

C 40.553829 38.116853 47.415750

C 41.312296 37.559195 46.295914

C 40.386328 36.933845 45.476699

C 39.072987 37.290142 46.035835

C 36.629001 37.753440 45.708315

C 35.476006 37.882740 44.821824

C 34.472906 38.484143 45.566341

C 35.054719 38.685198 46.904894

C 34.912252 39.215317 49.356208

C 34.121463 39.423548 50.564140

C 35.004235 39.366479 51.617364

C 36.333995 39.222868 51.031368

C 37.537942 39.336925 51.737938

H 37.464639 39.616684 52.790579

C 41.128301 38.624649 48.584189

H 42.214331 38.725535 48.613586

C 37.855143 37.172127 45.357072

H 37.884153 36.667353 44.387799

C 34.384986 39.118203 48.062714

H 33.313696 39.323984 47.971449

C 37.253608 42.751219 47.187407

C 38.613403 42.621400 46.828166

C 39.298391 43.671688 46.189811

C 38.608850 44.855244 45.973689

C 37.256609 44.976220 46.364584

C 36.554624 43.938952 46.964461

C 36.900986 41.494733 47.776855

H 40.341163 43.554601 45.898388

H 39.102510 45.709358 45.507637

H 35.515704 44.079667 47.258436

H 35.921992 41.218049 48.175609

N 36.559664 46.255468 46.162105

O 35.370008 46.305490 46.456231

O 37.202309 47.191090 45.718021

N 37.844546 40.612919 47.826029

O 39.108594 41.442589 47.151912

h 36.462088 34.846458 51.643113

h 40.207201 39.780513 52.966472

h 42.174684 39.368315 51.153198

h 42.369624 37.760535 46.123910

h 40.508998 36.263597 44.625943

h 35.562201 37.631840 43.764618

h 33.459297 38.801125 45.321010

h 33.047508 39.609735 50.559458

h 34.788645 39.276625 52.682030

**^3^IN1**

C 37.249416 35.372544 50.815239

H 38.072230 34.645922 50.838691

H 37.635045 36.273663 51.314376

S 36.759934 35.815305 49.107433

Fe 37.672838 38.378807 48.449742

N 39.033654 38.640456 49.913624

N 39.127334 37.694158 47.235945

N 36.321842 38.103450 46.959356

N 36.216697 38.938174 49.701611

C 38.802577 39.030179 51.214545

C 40.066746 39.294258 51.894343

C 41.059053 39.101090 50.961118

C 40.391949 38.710511 49.732543

C 40.470413 37.898171 47.392076

C 41.211627 37.359853 46.258368

C 40.290295 36.688130 45.475591

C 38.981066 37.023234 46.049940

C 36.575567 37.586467 45.723217

C 35.445425 37.804125 44.829731

C 34.466205 38.439595 45.577254

C 35.035106 38.578931 46.926053

C 34.900723 39.118184 49.373338

C 34.119915 39.374747 50.571588

C 34.996911 39.293603 51.632549

C 36.316750 39.085573 51.059497

C 37.526899 39.170417 51.763715

H 37.471910 39.496961 52.803880

C 41.056440 38.425768 48.541289

H 42.137501 38.566648 48.542716

C 37.767591 36.941994 45.367631

H 37.782461 36.455824 44.389691

C 34.376508 39.028698 48.077932

H 33.319825 39.290735 47.976730

C 37.138879 42.618875 47.200914

C 38.489098 43.012241 46.823494

C 38.634978 44.378469 46.356865

C 37.564458 45.235428 46.291347

C 36.263244 44.799491 46.668669

C 36.062521 43.500726 47.125835

C 36.975208 41.272321 47.697068

H 39.636936 44.712150 46.082224

H 37.691365 46.265582 45.956057

H 35.061447 43.200696 47.438016

H 35.943669 41.023416 48.011578

N 35.149231 45.714396 46.645143

O 34.022047 45.281032 46.910971

O 35.365655 46.892011 46.370703

N 37.863133 40.371670 47.807543

O 39.429175 42.194795 46.917955

h 36.540634 34.956456 51.531176

h 40.227023 39.610215 52.925143

h 42.136629 39.240440 51.047591

h 42.245143 37.624940 46.035569

h 40.414504 36.006410 44.634225

h 35.541494 37.591173 43.765077

h 33.473178 38.809641 45.322247

h 33.060673 39.631474 50.558812

h 34.775748 39.218978 52.697248

**^3^TS2**

C 37.268220 35.372251 50.799390

H 38.086376 34.641817 50.817666

H 37.655378 36.271889 51.298003

S 36.781202 35.816423 49.090044

Fe 37.672970 38.269105 48.410711

N 39.055645 38.613437 49.830312

N 39.113127 37.628450 47.170985

N 36.321839 38.037778 46.922348

N 36.265713 38.938031 49.652006

C 38.846959 38.961812 51.151945

C 40.124447 39.187149 51.818214

C 41.100416 39.018778 50.861509

C 40.415366 38.667573 49.634234

C 40.455114 37.859947 47.295730

C 41.180338 37.329201 46.151556

C 40.261666 36.614295 45.406406

C 38.956341 36.937334 45.995437

C 36.555926 37.506859 45.689958

C 35.434258 37.760614 44.796194

C 34.484890 38.446658 45.537656

C 35.064567 38.586435 46.881746

C 34.958283 39.169327 49.318880

C 34.181823 39.433078 50.517924

C 35.049514 39.299941 51.583020

C 36.364723 39.062147 51.013229

C 37.578772 39.107819 51.717231

H 37.536572 39.418982 52.763449

C 41.060424 38.396339 48.429657

H 42.140311 38.544948 48.407176

C 37.736832 36.842484 45.328916

H 37.741826 36.347174 44.355379

C 34.435609 39.098686 48.022944

H 33.398111 39.424777 47.915244

C 37.554968 42.693335 47.284205

C 38.623206 43.296087 46.481552

C 38.250228 44.544716 45.828446

C 36.994564 45.088850 45.924487

C 35.983250 44.421597 46.663574

C 36.276718 43.222694 47.332869

C 37.878191 41.484697 48.121690

H 39.032395 45.031968 45.240479

H 36.745693 46.029809 45.431598

H 35.487438 42.755023 47.922047

H 38.078560 41.617118 49.209471

N 34.666245 44.979863 46.758238

O 33.769011 44.302285 47.280943

O 34.464734 46.108098 46.306150

N 37.865166 40.338908 47.618541

O 39.757823 42.793723 46.374097

h 36.553894 34.957833 51.510770

h 40.311177 39.460587 52.856689

h 42.182058 39.134060 50.930982

h 42.201031 37.620851 45.904185

h 40.391681 35.911309 44.583622

h 35.526087 37.542647 43.732180

h 33.507361 38.852673 45.277551

h 33.128332 39.712454 50.504831

h 34.824450 39.227294 52.647038

**^3^IN2**

C 37.281603 35.378999 50.826348

H 38.106100 34.655666 50.856512

H 37.657886 36.285214 51.322297

S 36.807336 35.804930 49.108514

Fe 37.568331 38.334868 48.508508

N 38.960095 38.679958 49.918857

N 39.016118 37.795055 47.222925

N 36.204734 38.088960 47.022816

N 36.137645 38.853240 49.795374

C 38.749310 39.032319 51.237604

C 40.022586 39.326109 51.886426

C 40.997293 39.180217 50.926049

C 40.313496 38.793249 49.707300

C 40.361109 38.017367 47.355749

C 41.090532 37.476589 46.218428

C 40.166708 36.779884 45.460108

C 38.861817 37.102838 46.044581

C 36.442361 37.605409 45.772966

C 35.300713 37.841409 44.900384

C 34.330585 38.456383 45.677311

C 34.924480 38.580971 47.016802

C 34.829741 39.082947 49.473725

C 34.064751 39.347903 50.681296

C 34.947328 39.231830 51.733981

C 36.258516 39.003509 51.146671

C 37.484409 39.113789 51.823986

H 37.447074 39.439963 52.865326

C 40.958776 38.552118 48.496015

H 42.035647 38.721315 48.475016

C 37.639797 36.988599 45.385230

H 37.646515 36.514129 44.401238

C 34.294178 39.033621 48.182488

H 33.247252 39.335320 48.095294

C 37.687772 42.662136 46.867198

C 38.709135 43.432964 46.142396

C 38.288560 44.750121 45.692396

C 37.020351 45.223074 45.916015

C 36.056539 44.419612 46.586520

C 36.394532 43.151274 47.058909

C 38.163287 41.374039 47.353512

H 39.031688 45.349080 45.159859

H 36.721314 46.216899 45.580292

H 35.646614 42.569164 47.597250

H 39.256569 41.275228 47.152610

N 34.723493 44.928412 46.794647

O 33.857771 44.160159 47.225126

O 34.496539 46.107842 46.528156

N 37.548758 40.428178 47.904525

O 39.856662 42.984236 45.931375

h 36.568274 34.961228 51.536766

h 40.206081 39.649342 52.911080

h 42.073814 39.336506 50.994919

h 42.125548 37.731131 45.990356

h 40.286792 36.085521 44.628539

h 35.395184 37.665698 43.828811

h 33.326139 38.810409 45.445322

h 33.015363 39.642564 50.676177

h 34.736747 39.147337 52.800088

**^3^TS3**

C 37.258490 35.354365 50.808351

H 38.073887 34.619036 50.827155

H 37.656666 36.249993 51.308830

S 36.759134 35.804654 49.105354

Fe 37.610294 38.449062 48.443400

N 38.986846 38.719587 49.896334

N 39.062600 37.783066 47.211526

N 36.249593 38.145899 46.967239

N 36.161035 38.936899 49.726085

C 38.762211 39.078422 51.206154

C 40.030379 39.362591 51.872920

C 41.016195 39.195504 50.927484

C 40.342040 38.807325 49.701544

C 40.405109 37.998344 47.355586

C 41.141316 37.452139 46.222181

C 40.219395 36.762351 45.456794

C 38.910633 37.094314 46.036195

C 36.493435 37.625968 45.732709

C 35.353975 37.838530 44.849686

C 34.378584 38.470329 45.606995

C 34.965525 38.625086 46.947083

C 34.849015 39.137503 49.401934

C 34.070981 39.372202 50.609756

C 34.950038 39.263182 51.665526

C 36.269965 39.065184 51.081784

C 37.487703 39.169495 51.773295

H 37.437587 39.483398 52.817808

C 40.996003 38.538848 48.498519

H 42.076752 38.686153 48.489689

C 37.690910 36.993049 45.366996

H 37.700946 36.501400 44.391103

C 34.321393 39.078990 48.107193

H 33.267661 39.356503 48.014224

C 37.486598 42.772940 46.913858

C 38.526996 43.540848 46.275444

C 38.226691 44.849144 45.815393

C 36.950486 45.355877 46.005577

C 35.948651 44.579721 46.623557

C 36.205852 43.281609 47.084149

C 38.066614 41.476891 47.303516

H 39.003787 45.437409 45.322657

H 36.695709 46.364311 45.677485

H 35.419265 42.710994 47.576315

H 39.260046 41.875753 46.804629

N 34.619896 45.147731 46.800799

O 33.719221 44.402521 47.180451

O 34.459280 46.339359 46.564836

N 37.768306 40.409846 47.803111

O 39.683764 42.963665 46.181761

h 36.551835 34.943104 51.529163

h 40.198724 39.691305 52.898432

h 42.093919 39.335390 51.011207

h 42.175064 37.713781 45.996405

h 40.342121 36.069157 44.624636

h 35.448647 37.628529 43.784321

h 33.376062 38.822868 45.364647

h 33.013028 39.634434 50.603701

h 34.735811 39.174929 52.730601

**^3^PC1**

C 37.254731 35.222126 50.870014

H 38.059087 34.474009 50.928309

H 37.659968 36.125040 51.352099

S 36.771175 35.635736 49.157055

Fe 37.700917 38.107973 48.543925

N 39.053496 38.515914 49.994654

N 39.216554 37.567324 47.314050

N 36.422393 38.068938 46.964513

N 36.245338 38.878770 49.694131

C 38.791422 38.955366 51.270474

C 40.037856 39.247084 51.983512

C 41.056983 38.985504 51.102343

C 40.419635 38.548599 49.866391

C 40.554921 37.758532 47.511699

C 41.322826 37.313537 46.354120

C 40.412820 36.702621 45.500224

C 39.096773 36.974124 46.085373

C 36.683991 37.540077 45.731043

C 35.573486 37.777779 44.808273

C 34.600437 38.448206 45.527967

C 35.151408 38.581586 46.891826

C 34.947496 39.109352 49.335779

C 34.130478 39.375400 50.513571

C 34.974255 39.261349 51.597179

C 36.305072 39.022385 51.053371

C 37.500739 39.108482 51.783684

H 37.422266 39.454813 52.816575

C 41.114230 38.238020 48.700396

H 42.200959 38.331581 48.741988

C 37.884226 36.907077 45.386784

H 37.914663 36.435443 44.400702

C 34.468074 39.051357 48.022396

H 33.422730 39.347370 47.887978

C 37.668358 43.180512 46.635003

C 38.583370 43.821797 45.753463

C 38.259693 45.088917 45.227899

C 37.053526 45.691112 45.550632

C 36.137102 45.016557 46.373928

C 36.432372 43.774867 46.924927

C 38.058679 41.933725 47.218870

H 38.985470 45.577465 44.573693

H 36.802491 46.681276 45.170388

H 35.721893 43.283484 47.588087

H 39.914592 42.426041 45.824812

N 34.833883 45.628800 46.661699

O 33.960365 44.912436 47.133448

O 34.693567 46.814629 46.408176

N 38.454279 40.931744 47.653050

O 39.745796 43.282135 45.397379

h 36.530563 34.841247 51.590137

h 40.159547 39.654025 52.987333

h 42.133187 39.112228 51.219785

h 42.359749 37.597418 46.174487

h 40.561051 36.092003 44.609584

h 35.674552 37.543216 43.748635

h 33.626566 38.852191 45.251490

h 33.074957 39.644837 50.476908

h 34.732837 39.184568 52.657315

**^1^RC1**

C 37.269226 35.313234 50.872393

H 38.105117 34.604698 50.961359

H 37.613153 36.245715 51.339413

S 36.848148 35.671984 49.125107

Fe 37.687054 37.933781 48.512152

N 39.052446 38.442812 49.935758

N 39.211552 37.431921 47.265357

N 36.445318 37.930969 46.906521

N 36.267811 38.813110 49.632697

C 38.806357 38.909226 51.209443

C 40.060826 39.206073 51.909559

C 41.067600 38.937419 51.016416

C 40.417172 38.475887 49.794919

C 40.549897 37.634363 47.453024

C 41.321170 37.151245 46.310645

C 40.424087 36.493945 45.488576

C 39.101861 36.797190 46.056452

C 36.719487 37.425477 45.667200

C 35.648497 37.729439 44.718732

C 34.673605 38.405523 45.433672

C 35.186550 38.477118 46.818897

C 34.966445 39.022815 49.265143

C 34.146715 39.311696 50.435909

C 34.989978 39.236684 51.524672

C 36.322875 38.993544 50.989309

C 37.518562 39.087280 51.721137

H 37.442289 39.457971 52.746365

C 41.107252 38.137616 48.631538

H 42.192340 38.246137 48.666735

C 37.903346 36.748476 45.341158

H 37.940400 36.278438 44.354031

C 34.495582 38.943243 47.947715

H 33.452592 39.244226 47.802543

C 37.409441 43.418028 46.918852

C 38.398668 44.155694 46.243213

C 38.176000 45.431818 45.707746

C 36.899365 45.951525 45.876544

C 35.897189 45.201345 46.535492

C 36.119773 43.936060 47.067204

C 38.104249 42.220552 47.301418

H 38.968642 45.984742 45.200179

H 36.646879 46.945969 45.509074

H 35.324027 43.403686 47.585388

H 37.741887 41.358186 47.864731

N 34.553974 45.787916 46.685635

O 33.663585 45.065746 47.115192

O 34.401298 46.958772 46.379224

N 39.337423 42.246271 46.883246

O 39.538661 43.450502 46.211001

h 36.546059 34.906068 51.579013

h 40.194017 39.584211 52.923136

h 42.147027 39.054466 51.112361

h 42.345451 37.459959 46.101814

h 40.573545 35.843860 44.626532

h 35.806028 37.536512 43.657586

h 33.730467 38.867681 45.142183

h 33.087927 39.566870 50.392084

h 34.750799 39.192169 52.587159

**^1^TS1**

C 37.342331 35.460457 50.765834

H 38.143664 34.712445 50.785186

H 37.749141 36.356830 51.252244

S 36.885222 35.902573 49.042989

Fe 37.566800 38.221651 48.510743

N 38.920017 38.542832 49.989522

N 39.056401 37.610393 47.281600

N 36.246396 37.998433 46.973604

N 36.098750 38.766382 49.754155

C 38.681164 38.977858 51.270976

C 39.943043 39.256367 51.965641

C 40.942075 38.998858 51.060427

C 40.278791 38.576007 49.833582

C 40.395457 37.776394 47.483183

C 41.166670 37.279303 46.344228

C 40.255815 36.674524 45.498204

C 38.937176 36.998687 46.061740

C 36.531941 37.567855 45.714704

C 35.417690 37.823852 44.806053

C 34.404477 38.376289 45.573417

C 34.948715 38.435725 46.946209

C 34.781615 38.913516 49.414898

C 33.987370 39.199656 50.602824

C 34.861712 39.178392 51.667079

C 36.189591 38.973031 51.102379

C 37.397434 39.119738 51.805232

H 37.330230 39.486335 52.832186

C 40.961030 38.260467 48.661761

H 42.045827 38.365790 48.694223

C 37.739539 36.957744 45.348171

H 37.778586 36.527810 44.344119

C 34.266961 38.815604 48.113727

H 33.203204 39.050816 48.011393

C 37.072366 42.354654 47.322511

C 38.477994 42.398724 47.074150

C 39.027244 43.605567 46.550049

C 38.202582 44.697303 46.341943

C 36.818226 44.619123 46.617663

C 36.242867 43.448674 47.107554

C 36.740935 41.046419 47.825053

H 40.093803 43.670224 46.335125

H 38.611003 45.636677 45.967591

H 35.176226 43.423609 47.329305

H 35.703847 40.802404 48.092296

N 35.969281 45.788592 46.431490

O 34.756737 45.655328 46.598593

O 36.500482 46.846821 46.123373

N 37.655842 40.168542 47.952936

O 39.117712 41.319511 47.352482

h 36.619877 35.058193 51.475982

h 40.095692 39.620520 52.981593

h 42.023311 39.102120 51.151705

h 42.213122 37.523382 46.161391

h 40.389198 36.041482 44.620974

h 35.571810 37.693743 43.734893

h 33.410852 38.748478 45.323916

h 32.926763 39.449630 50.576494

h 34.644086 39.124823 52.733773

**^1^IN1**

C 37.317224 35.498024 50.773868

H 38.152069 34.788176 50.821819

H 37.670709 36.417128 51.258788

S 36.885745 35.904384 49.036578

Fe 37.559802 38.217408 48.505201

N 38.931919 38.540445 49.968598

N 39.032902 37.616001 47.253694

N 36.225114 37.967507 46.990528

N 36.114524 38.755801 49.775624

C 38.711805 38.966750 51.257141

C 39.981637 39.247911 51.931614

C 40.966274 39.016962 51.003603

C 40.286656 38.599393 49.784645

C 40.372273 37.809512 47.431291

C 41.131701 37.309676 46.287614

C 40.218548 36.673265 45.468167

C 38.903340 36.989670 46.042097

C 36.491207 37.532125 45.727626

C 35.355379 37.770591 44.843071

C 34.360738 38.337345 45.622745

C 34.931804 38.418752 46.980350

C 34.800584 38.934512 49.442698

C 34.025155 39.258951 50.630993

C 34.905866 39.224372 51.689472

C 36.222752 38.980155 51.119563

C 37.438015 39.117900 51.809426

H 37.386736 39.499132 52.831642

C 40.951249 38.308132 48.596869

H 42.033296 38.441780 48.606220

C 37.697120 36.931836 45.343468

H 37.726702 36.497671 44.341061

C 34.272238 38.827687 48.149056

H 33.212092 39.080227 48.055470

C 37.114793 42.327272 47.326598

C 38.525880 42.548661 47.100633

C 38.891430 43.866849 46.643534

C 37.943651 44.851499 46.469491

C 36.572842 44.586136 46.713386

C 36.164613 43.326574 47.145004

C 36.788758 41.003381 47.797403

H 39.946072 44.081390 46.469060

H 38.236773 45.852980 46.151855

H 35.108744 43.156964 47.356295

H 35.720392 40.820684 48.013339

N 35.586799 45.636406 46.584409

O 34.397633 45.349063 46.752974

O 35.973577 46.770631 46.321550

N 37.596517 40.047823 47.979568

O 39.334191 41.604664 47.317392

h 36.597907 35.073966 51.474457

h 40.147166 39.598251 52.950402

h 42.046185 39.143250 51.080486

h 42.173063 37.561971 46.087669

h 40.350757 36.023418 44.603131

h 35.464163 37.620382 43.768981

h 33.359443 38.698286 45.387806

h 32.975493 39.551713 50.607223

h 34.695258 39.175551 52.757801

**^1^TS2**

C 37.319690 35.430286 50.774803

H 38.133716 34.697615 50.806686

H 37.702713 36.338526 51.256213

S 36.877228 35.842183 49.042136

Fe 37.569534 38.080750 48.468412

N 38.971781 38.413034 49.903300

N 39.005243 37.448718 47.202841

N 36.227602 37.889285 46.969298

N 36.173979 38.716779 49.745662

C 38.783949 38.821333 51.207281

C 40.074715 39.066322 51.856850

C 41.034289 38.841122 50.900313

C 40.323545 38.447956 49.692632

C 40.350307 37.631283 47.346826

C 41.078526 37.089990 46.203727

C 40.146764 36.426106 45.432473

C 38.844957 36.779584 46.016367

C 36.456351 37.401766 45.719847

C 35.336833 37.697555 44.832074

C 34.390254 38.356740 45.598836

C 34.972421 38.440520 46.951432

C 34.874546 38.981008 49.408985

C 34.114291 39.315128 50.603946

C 34.989173 39.210254 51.665092

C 36.294127 38.914037 51.093625

C 37.521086 38.998756 51.778220

H 37.492701 39.370279 52.805552

C 40.959933 38.140010 48.491496

H 42.045359 38.241727 48.480927

C 37.628209 36.730722 45.339807

H 37.625833 36.264547 44.351468

C 34.349171 38.920072 48.111517

H 33.314035 39.258140 48.012654

C 37.315313 42.367710 47.382758

C 38.464853 42.706759 46.538752

C 38.336324 43.973889 45.830366

C 37.224897 44.773551 45.925491

C 36.127762 44.375010 46.732962

C 36.200778 43.192538 47.476020

C 37.363275 41.146758 48.230860

H 39.185298 44.266203 45.207670

H 37.158927 45.716673 45.380742

H 35.355594 42.929509 48.111584

H 37.265704 41.311680 49.329167

N 34.934576 45.174557 46.790307

O 33.925702 44.696065 47.325683

O 34.954788 46.300019 46.294905

N 37.675197 39.974698 47.918622

O 39.480066 41.992198 46.467283

h 36.592388 35.023151 51.477185

h 40.280145 39.380440 52.880175

h 42.118911 38.939718 50.944342

h 42.111064 37.342714 45.962767

h 40.258749 35.740013 44.592948

h 35.442924 37.523003 43.761400

h 33.414225 38.776211 45.354956

h 33.071354 39.631440 50.586746

h 34.772393 39.155636 52.731905

**^1^IN2**

C 37.332491 35.492826 50.769607

H 38.127328 34.739205 50.766298

H 37.749527 36.380640 51.261435

S 36.863187 35.945895 49.054560

Fe 37.533265 38.205347 48.554480

N 38.926951 38.487736 50.016251

N 38.986937 37.591878 47.280326

N 36.208471 38.042404 47.031136

N 36.126986 38.821130 49.817814

C 38.722031 38.930870 51.303058

C 40.001394 39.232322 51.948772

C 40.973243 39.002336 51.005569

C 40.278271 38.565152 49.803623

C 40.326721 37.809585 47.432859

C 41.067009 37.336911 46.268201

C 40.150114 36.679779 45.469427

C 38.841508 36.972163 46.068032

C 36.444488 37.567385 45.775653

C 35.312517 37.838840 44.896516

C 34.351098 38.464662 45.672104

C 34.941010 38.565035 47.018776

C 34.830663 39.084960 49.476502

C 34.055552 39.389982 50.670682

C 34.921079 39.266638 51.735449

C 36.233744 38.990771 51.169523

C 37.451269 39.083073 51.865065

H 37.406277 39.452903 52.891659

C 40.920951 38.300772 48.595304

H 42.001171 38.449657 48.582390

C 37.625812 36.923035 45.385622

H 37.632920 36.484888 44.384688

C 34.308790 39.030820 48.179256

H 33.275119 39.369413 48.075881

C 37.568344 42.267733 47.103923

C 38.490948 43.225584 46.479939

C 37.892769 44.485364 46.076246

C 36.556876 44.743672 46.260515

C 35.701307 43.762722 46.832841

C 36.207359 42.530304 47.246047

C 38.247404 41.069633 47.568864

H 38.555383 45.219998 45.611895

H 36.120385 45.697754 45.960905

H 35.536405 41.806986 47.708931

H 39.352662 41.213923 47.446135

N 34.301645 44.048302 47.011115

O 33.546587 43.116657 47.318788

O 33.905655 45.199147 46.837230

N 37.848245 39.981064 48.029548

O 39.703705 42.960379 46.317101

h 36.609492 35.085929 51.476553

h 40.185624 39.623195 52.949444

h 42.052522 39.144308 51.060945

h 42.090275 37.634730 46.039514

h 40.274609 36.029792 44.603352

h 35.410554 37.679005 43.822781

h 33.353268 38.836016 45.438656

h 33.014978 39.713487 50.645824

h 34.698038 39.187060 52.799398

**^1^TS3**

C 37.349124 35.456373 50.768768

H 38.146113 34.703852 50.779701

H 37.762327 36.346916 51.260124

S 36.883626 35.911078 49.052360

Fe 37.585115 38.213040 48.518701

N 38.964592 38.487999 49.992491

N 39.044586 37.564348 47.266208

N 36.260525 38.014316 46.996976

N 36.161615 38.801633 49.783483

C 38.750860 38.924689 51.278473

C 40.026413 39.222323 51.936793

C 41.004897 38.986104 51.002783

C 40.317349 38.552015 49.794340

C 40.382879 37.777006 47.427880

C 41.132484 37.298970 46.269638

C 40.219476 36.649421 45.461800

C 38.906073 36.945969 46.053809

C 36.505261 37.536556 45.745278

C 35.374912 37.798192 44.859064

C 34.404888 38.418416 45.628890

C 34.989108 38.525009 46.979044

C 34.862208 39.040961 49.440119

C 34.076396 39.328968 50.633588

C 34.941781 39.224622 51.700035

C 36.261419 38.971690 51.134599

C 37.476345 39.075416 51.833384

H 37.426258 39.444325 52.860332

C 40.969573 38.269496 48.594259

H 42.053227 38.396152 48.594712

C 37.693613 36.896942 45.364466

H 37.708637 36.452502 44.366075

C 34.347268 38.982251 48.139195

H 33.303821 39.292596 48.035847

C 37.336045 42.292580 47.187311

C 38.325256 43.210468 46.677794

C 37.898537 44.494171 46.245770

C 36.559612 44.835737 46.351630

C 35.617469 43.915288 46.854507

C 35.994405 42.632055 47.272028

C 38.042184 41.063837 47.570977

H 38.632851 45.194752 45.843129

H 36.207338 45.821613 46.045803

H 35.248950 41.943974 47.668155

H 39.200602 41.597979 47.229952

N 34.226113 44.312660 46.970120

O 33.401631 43.443155 47.256345

O 33.931919 45.486020 46.775136

N 37.839047 39.939452 47.982006

O 39.546506 42.783905 46.665204

h 36.625366 35.053988 51.477518

h 40.202500 39.605896 52.941746

h 42.085199 39.117923 51.063110

h 42.156455 37.600703 46.049396

h 40.352665 36.004052 44.593568

h 35.473863 37.626664 43.787218

h 33.407062 38.786835 45.390817

h 33.028315 39.627241 50.608574

h 34.718303 39.152154 52.764400

**^1^PC1**

C 37.315918 35.413799 50.740181

H 38.110617 34.657239 50.757962

H 37.738664 36.306712 51.221701

S 36.827229 35.863594 49.028969

Fe 37.454481 38.241810 48.473470

N 38.837090 38.589107 49.936535

N 38.963064 37.672007 47.224289

N 36.131399 38.013724 46.948787

N 36.000425 38.788252 49.716060

C 38.599517 39.002045 51.222729

C 39.862421 39.308091 51.910378

C 40.860469 39.076311 50.996272

C 40.193963 38.651812 49.769139

C 40.300857 37.867527 47.404462

C 41.070519 37.356638 46.268381

C 40.159684 36.719785 45.447149

C 38.839894 37.033982 46.021627

C 36.414684 37.564550 45.694701

C 35.298246 37.814612 44.783481

C 34.290895 38.387861 45.544419

C 34.844534 38.478129 46.913869

C 34.694363 38.993863 49.377840

C 33.904905 39.283414 50.573342

C 34.775025 39.207621 51.637282

C 36.099427 38.973387 51.064822

C 37.312074 39.111170 51.763689

H 37.246035 39.455752 52.798697

C 40.867468 38.367895 48.580610

H 41.951972 38.489348 48.604044

C 37.629410 36.965164 45.325526

H 37.663535 36.524806 44.324667

C 34.181442 38.914844 48.074612

H 33.126437 39.188843 47.968485

C 37.234047 42.504560 47.150253

C 38.407607 43.144871 46.671434

C 38.342050 44.439245 46.138078

C 37.130837 45.122964 46.138939

C 35.985725 44.497451 46.651335

C 36.014857 43.195979 47.143379

C 37.366492 41.160983 47.615258

H 39.255578 44.903676 45.760907

H 37.059731 46.144389 45.764110

H 35.108096 42.737219 47.532650

H 39.537421 41.796095 47.388939

N 34.720422 45.246913 46.713368

O 33.713908 44.636076 47.045686

O 34.750575 46.436010 46.442628

N 37.543325 40.056435 47.939761

O 39.591677 42.509158 46.727488

h 36.600749 35.021083 51.462937

h 40.012846 39.686353 52.921494

h 41.941511 39.183514 51.085275

h 42.116332 37.598697 46.079309

h 40.291144 36.066076 44.584914

h 35.454934 37.678814 43.713399

h 33.289817 38.741203 45.297320

h 32.852418 39.566012 50.551492

h 34.564936 39.136784 52.704480

**^5^RC1’**

C 34.556921 44.153028 50.378422

C 35.095126 45.419441 50.087670

H 34.454517 46.242787 49.751897

C 36.469081 45.645461 50.200582

H 36.884662 46.626610 49.953165

C 37.318201 44.619588 50.627382

H 38.387222 44.807353 50.726612

C 35.420383 43.125349 50.792970

H 35.047719 42.119280 51.017366

C 36.794683 43.358624 50.922906

H 37.450599 42.553792 51.259505

C 37.270173 35.171883 50.940501

H 38.094435 34.453991 51.055569

H 37.627859 36.111533 51.383317

S 36.849652 35.478276 49.185833

Fe 37.679758 37.687960 48.573968

N 39.121180 38.365576 49.997236

N 39.277692 37.398392 47.229611

N 36.408284 37.840490 46.855771

N 36.266212 38.824940 49.633761

C 38.852788 38.867695 51.244208

C 40.110804 39.169162 51.948227

C 41.123515 38.875690 51.066178

C 40.477650 38.393744 49.844543

C 40.607901 37.583121 47.457197

C 41.385159 37.146196 46.299429

C 40.488676 36.542885 45.430482

C 39.159053 36.825502 45.993352

C 36.720276 37.392408 45.610865

C 35.642236 37.703925 44.664815

C 34.651613 38.345467 45.394103

C 35.159198 38.392536 46.788138

C 34.965028 38.999345 49.257459

C 34.148973 39.289665 50.433216

C 34.999088 39.233219 51.522563

C 36.337476 39.002394 50.986904

C 37.547432 39.082473 51.719303

H 37.464586 39.469785 52.738600

C 41.153787 38.046161 48.668225

H 42.241162 38.134329 48.710471

C 37.946899 36.778534 45.280408

H 38.005099 36.346053 44.276900

C 34.490507 38.875989 47.933790

H 33.444890 39.173773 47.795889

C 37.454701 43.617468 46.761803

C 38.417326 44.355072 46.048529

C 38.175771 45.631092 45.521623

C 36.903612 46.147083 45.732158

C 35.925382 45.395217 46.424530

C 36.171124 44.135691 46.959329

C 38.161114 42.416823 47.110716

H 38.950966 46.184119 44.987417

H 36.636246 47.140995 45.373673

H 35.396810 43.606849 47.512299

H 37.818869 41.548476 47.676076

N 34.582908 45.972078 46.605794

O 33.706288 45.243441 47.053972

O 34.415091 47.142506 46.305759

N 39.376885 42.444057 46.644567

O 39.554469 43.648753 45.971083

O 41.541841 40.497988 46.149727

H 41.179732 39.723520 46.601362

H 40.894271 41.201376 46.344123

h 36.528908 34.797391 51.646438

h 40.235903 39.563612 52.956605

h 42.202687 38.996484 51.160320

h 42.408241 37.471618 46.111091

h 40.648883 35.929479 44.543840

h 35.801621 37.531534 43.600416

h 33.704059 38.804949 45.112885

h 33.088390 39.537925 50.393255

h 34.758209 39.183713 52.584445

h 33.488217 43.969102 50.268383

**^3^RC1’**

C 34.553854 44.104859 50.375075

C 35.122160 45.361344 50.099419

H 34.501218 46.204294 49.775876

C 36.501444 45.552576 50.212987

H 36.941722 46.525632 49.976553

C 37.325445 44.502287 50.629025

H 38.398171 44.663928 50.730521

C 35.392846 43.051647 50.775989

H 34.997186 42.051998 50.989955

C 36.772117 43.250685 50.909243

H 37.410581 42.429080 51.238235

C 37.262139 35.222010 50.920256

H 38.116134 34.542732 51.062819

H 37.568336 36.179101 51.367251

S 36.888137 35.504260 49.152633

Fe 37.720410 37.898694 48.515720

N 39.076306 38.409315 49.939985

N 39.229882 37.408053 47.262005

N 36.467750 37.889078 46.907167

N 36.287049 38.728259 49.651476

C 38.827715 38.853114 51.220225

C 40.081017 39.142190 51.921907

C 41.088642 38.888520 51.023139

C 40.440088 38.445485 49.795751

C 40.566475 37.618871 47.449104

C 41.336093 37.139930 46.306002

C 40.443986 36.467899 45.489959

C 39.121016 36.766456 46.056236

C 36.741611 37.390138 45.666119

C 35.670581 37.699695 44.720833

C 34.697559 38.374553 45.441295

C 35.208949 38.434265 46.826131

C 34.987681 38.944750 49.280607

C 34.169291 39.235260 50.451461

C 35.009819 39.156398 51.542004

C 36.343156 38.912809 51.007144

C 37.539298 39.016667 51.737043

H 37.461833 39.376458 52.765653

C 41.126085 38.121617 48.626336

H 42.210996 38.232127 48.658330

C 37.923128 36.709231 45.340304

H 37.959502 36.235133 44.355592

C 34.517549 38.883424 47.961606

H 33.475120 39.186817 47.819440

C 37.456946 43.656514 46.736988

C 38.413581 44.402446 46.024405

C 38.166553 45.683257 45.511925

C 36.893787 46.193412 45.732473

C 35.920563 45.432578 46.422414

C 36.173059 44.169729 46.946162

C 38.168324 42.454754 47.070452

H 38.937946 46.243805 44.980345

H 36.622211 47.188735 45.381237

H 35.402123 43.635074 47.498343

H 37.830040 41.581266 47.629786

N 34.574766 46.000586 46.607357

O 33.704899 45.266942 47.060322

O 34.397796 47.168837 46.304469

N 39.380738 42.487798 46.595797

O 39.551778 43.698907 45.933697

O 41.534630 40.516692 46.149310

H 41.148911 39.756770 46.606621

H 40.902826 41.237653 46.331442

h 36.532645 34.820860 51.623807

h 40.214223 39.513138 52.938135

h 42.168487 39.001576 51.119152

h 42.352847 37.467991 46.090005

h 40.598400 35.812977 44.632463

h 35.829000 37.517990 43.657841

h 33.756745 38.842734 45.151910

h 33.112096 39.496858 50.407040

h 34.766983 39.115931 52.603823

h 33.480783 43.947578 50.266225

**^3^TS1’**

C 34.558360 44.333125 50.325809

C 34.992991 45.568235 49.808917

H 34.292408 46.264492 49.334679

C 36.345893 45.918341 49.848264

H 36.674794 46.869108 49.419242

C 37.281599 45.041456 50.405992

H 38.339808 45.310840 50.417693

C 35.506068 43.467033 50.897282

H 35.219021 42.489804 51.303321

C 36.859853 43.821371 50.940938

H 37.580222 43.134621 51.387911

C 37.261762 35.290790 50.835480

H 38.078477 34.556724 50.884376

H 37.652301 36.196793 51.322882

S 36.782500 35.710209 49.120787

Fe 37.692416 38.274865 48.464482

N 39.033199 38.589829 49.950306

N 39.196750 37.626490 47.282832

N 36.376431 38.040877 46.922975

N 36.200440 38.848906 49.661678

C 38.765990 38.994903 51.233422

C 40.011701 39.281116 51.950380

C 41.031005 39.063683 51.054698

C 40.395769 38.650268 49.812312

C 40.530330 37.834188 47.473977

C 41.307280 37.336082 46.342754

C 40.410574 36.681463 45.517397

C 39.083745 36.987218 46.077746

C 36.673921 37.544056 45.689154

C 35.577417 37.779320 44.755067

C 34.567420 38.397706 45.479343

C 35.087173 38.499331 46.855290

C 34.887369 39.013714 49.311542

C 34.080817 39.284343 50.495048

C 34.940013 39.221397 51.569915

C 36.274040 39.014321 51.017541

C 37.470509 39.125496 51.744358

H 37.385286 39.455962 52.781770

C 41.088941 38.352374 48.642104

H 42.170791 38.490810 48.665310

C 37.887106 36.916643 45.362636

H 37.935399 36.441085 44.379483

C 34.393610 38.927314 48.001624

H 33.335195 39.177085 47.878245

C 37.168631 42.484461 47.220921

C 38.566550 42.640675 47.000067

C 39.050851 43.889840 46.529987

C 38.152594 44.924795 46.334503

C 36.771905 44.739215 46.572789

C 36.263678 43.523402 47.017936

C 36.945257 41.136683 47.676279

H 40.116575 44.040287 46.364190

H 38.502916 45.902909 46.002994

H 35.198090 43.419987 47.217251

H 35.940118 40.771269 47.918867

N 35.851122 45.859877 46.404215

O 34.645240 45.626803 46.455268

O 36.325532 46.973687 46.233682

N 37.942924 40.344030 47.795130

O 39.270114 41.587427 47.266657

O 41.450120 40.546183 45.858507

H 41.078321 39.663797 45.724886

H 40.790076 40.972179 46.437250

h 36.546262 34.891178 51.554115

h 40.135439 39.630459 52.975424

h 42.108171 39.184621 51.169394

h 42.337646 37.639837 46.157970

h 40.566465 36.027412 44.659503

h 35.725720 37.579824 43.693807

h 33.594412 38.801311 45.199297

h 33.019374 39.530263 50.464563

h 34.709550 39.163422 52.633678

h 33.509508 44.048192 50.243418

**^3^IN1’**

C 34.549786 44.285070 50.388514

C 35.019351 45.510956 49.882180

H 34.342328 46.221847 49.396072

C 36.379921 45.824782 49.933486

H 36.738742 46.765488 49.506784

C 37.287318 44.922517 50.497783

H 38.351270 45.164791 50.515509

C 35.468729 43.392670 50.965488

H 35.154492 42.418774 51.360378

C 36.830652 43.711539 51.023242

H 37.530995 43.000619 51.464573

C 37.321399 35.373861 50.782923

H 38.125088 34.628270 50.796877

H 37.729939 36.268324 51.271745

S 36.826635 35.821382 49.074702

Fe 37.548510 38.207762 48.500375

N 38.941343 38.481732 49.941435

N 39.019015 37.555981 47.265494

N 36.193372 37.961645 47.014045

N 36.107337 38.752151 49.753718

C 38.723786 38.883698 51.236455

C 39.996245 39.174134 51.903028

C 40.976748 38.987094 50.959416

C 40.296141 38.578678 49.741595

C 40.355285 37.784204 47.401144

C 41.095091 37.282950 46.248655

C 40.175743 36.611279 45.465928

C 38.867714 36.916684 46.066026

C 36.459258 37.496318 45.758808

C 35.336303 37.750722 44.865009

C 34.345026 38.344829 45.631571

C 34.907033 38.436334 46.987456

C 34.791339 38.957159 49.438622

C 34.033166 39.262988 50.640641

C 34.921133 39.180837 51.690527

C 36.231040 38.932377 51.104373

C 37.450298 39.028915 51.794035

H 37.404997 39.386944 52.824906

C 40.948767 38.318040 48.542418

H 42.022672 38.503265 48.515610

C 37.653369 36.865848 45.381576

H 37.669259 36.418942 44.384487

C 34.253257 38.871473 48.149816

H 33.198496 39.145493 48.057138

C 37.226072 42.302653 47.280232

C 38.627154 42.596172 47.027770

C 38.920561 43.929404 46.550713

C 37.930636 44.868300 46.395652

C 36.574306 44.540941 46.673143

C 36.229455 43.267798 47.109190

C 36.919048 40.971332 47.731946

H 39.962658 44.186722 46.357893

H 38.167841 45.881815 46.070022

H 35.184280 43.051148 47.332408

H 35.849343 40.779280 47.934473

N 35.547343 45.552822 46.570673

O 34.368804 45.214091 46.716816

O 35.891531 46.712277 46.361242

N 37.738911 40.013087 47.908375

O 39.489019 41.706132 47.235256

O 41.531236 40.586380 45.938593

H 41.016149 39.773290 45.862539

H 40.914979 41.165666 46.440648

h 36.601609 34.971116 51.495500

h 40.169482 39.507961 52.926070

h 42.056225 39.124275 51.022476

h 42.121147 37.570128 46.018865

h 40.304592 35.943288 44.614311

h 35.452882 37.592179 43.792936

h 33.348102 38.711330 45.386854

h 32.983190 39.555479 50.632453

h 34.712562 39.115174 52.758353

h 33.496099 44.024794 50.288189

**^1^RC-Zn**

C 37.267604 35.230918 50.939551

H 38.112158 34.539691 51.072701

H 37.587301 36.183845 51.382483

S 36.866938 35.495970 49.175464

ZN 37.681311 37.736534 48.569999

N 39.107329 38.484573 49.966383

N 39.280239 37.471825 47.223209

N 36.429853 37.953232 46.841951

N 36.259586 38.886173 49.626038

C 38.836341 38.956043 51.222547

C 40.089251 39.243181 51.935673

C 41.106533 38.957386 51.054208

C 40.463974 38.493110 49.825913

C 40.609831 37.654300 47.450647

C 41.392822 37.200408 46.298738

C 40.495231 36.600582 45.431074

C 39.164898 36.896109 45.992536

C 36.733019 37.478369 45.606533

C 35.643947 37.755214 44.665556

C 34.649943 38.396078 45.392638

C 35.169577 38.473584 46.778748

C 34.960235 39.052643 49.250475

C 34.133333 39.324751 50.427778

C 34.981082 39.274320 51.516375

C 36.323476 39.059806 50.977502

C 37.529804 39.145539 51.708920

H 37.446731 39.515107 52.734545

C 41.149866 38.134584 48.655733

H 42.236984 38.222257 48.705217

C 37.953999 36.849289 45.280354

H 38.006414 36.401833 44.283469

C 34.489775 38.940100 47.926581

H 33.438188 39.215781 47.788524

C 37.412580 43.405944 46.915479

C 38.407508 44.145544 46.250128

C 38.193048 45.426477 45.723200

C 36.918313 45.950913 45.889776

C 35.910271 45.200337 46.539506

C 36.125052 43.929846 47.062493

C 38.100936 42.201691 47.290593

H 38.990702 45.979057 45.222984

H 36.671559 46.948992 45.527992

H 35.324900 43.397740 47.574027

H 37.733635 41.332811 47.840044

N 34.570046 45.792885 46.689947

O 33.674717 45.073493 47.113851

O 34.423903 46.966304 46.389388

N 39.336168 42.227648 46.878786

O 39.544715 43.436851 46.217280

h 36.530502 34.839391 51.640588

h 40.213237 39.624803 52.949112

h 42.185262 39.071110 51.161338

h 42.424934 37.499945 46.116832

h 40.649028 35.981726 44.547089

h 35.792146 37.556944 43.604051

h 33.692943 38.836062 45.112223

h 33.069242 39.556947 50.384627

h 34.741247 39.220876 52.578302

**^3^RC-Zn**

C 37.251364 35.303573 50.934311

H 38.124971 34.651011 51.070808

H 37.523418 36.271903 51.379093

S 36.859154 35.552008 49.167602

ZN 37.698266 37.770183 48.568675

N 39.116941 38.486465 49.990122

N 39.291468 37.519352 47.208158

N 36.426769 37.956657 46.843532

N 36.264099 38.907698 49.638906

C 38.853727 38.953603 51.243928

C 40.105683 39.239732 51.957192

C 41.124482 38.960269 51.073052

C 40.488410 38.505042 49.844807

C 40.632413 37.707575 47.434748

C 41.409275 37.237616 46.316423

C 40.497142 36.634312 45.432120

C 39.188906 36.917044 45.992228

C 36.719835 37.475801 45.613277

C 35.632823 37.757761 44.668914

C 34.642757 38.410205 45.392495

C 35.156962 38.491928 46.774868

C 34.947922 39.060777 49.263125

C 34.125900 39.298964 50.421653

C 34.981707 39.246257 51.526081

C 36.309364 39.053345 50.991373

C 37.540887 39.140969 51.732104

H 37.458466 39.536569 52.747933

C 41.156700 38.178577 48.668851

H 42.244236 38.274981 48.714819

C 37.937908 36.826082 45.296399

H 37.980457 36.355319 44.309545

C 34.496780 38.960127 47.920111

H 33.448700 39.251482 47.779631

C 37.414012 43.377170 46.929064

C 38.418997 44.095524 46.255808

C 38.222137 45.374404 45.716636

C 36.955405 45.919192 45.879630

C 35.937501 45.189577 46.537712

C 36.134476 43.921225 47.072319

C 38.084563 42.166346 47.314736

H 39.026733 45.911257 45.210431

H 36.722854 46.917507 45.508908

H 35.327080 43.406222 47.589668

H 37.702643 41.309555 47.872861

N 34.606423 45.803178 46.685434

O 33.699036 45.099311 47.110400

O 34.479480 46.977843 46.381715

N 39.319715 42.168746 46.901816

O 39.545227 43.369116 46.228193

h 36.523951 34.883013 51.628620

h 40.229148 39.623405 52.969920

h 42.202544 39.078784 51.181692

h 42.445545 37.522188 46.134132

h 40.653977 36.015849 44.548393

h 35.789139 37.560321 43.608419

h 33.689481 38.854627 45.106453

h 33.061490 39.530629 50.383841

h 34.738361 39.191482 52.587142
